# Fixed-time event-triggered control for multi-agent systems with input delay

**DOI:** 10.1371/journal.pone.0293424

**Published:** 2023-11-13

**Authors:** Linling Wang, Xiaoyan Xu, Bing Han

**Affiliations:** 1 The Shanghai Engineering Research Center of Intelligent Maritime Search & Rescue and Underwater Vehicles, Shanghai Maritime University, Shanghai, China; 2 College of Physics and Electronic Information Engineering, Minjiang University, Fuzhou, China; 3 Shanghai Ship and Shipping Research Institute Co., Ltd., Shanghai, China; University of Shanghai for Science and Technology, CHINA

## Abstract

An in-depth study on the fixed-time event-triggered obstacle avoidance consensus control in heterogeneous USV-AUV systems with input delay and uncertain disturbances are conducted in this paper. When initial state of the system fails to achieve consensus, the desired heterogeneous USV-AUV formation can be achieved by fixed-time consensus control, within a fixed predetermined time, regardless of the initial states. Besides, an event-triggered communication strategy among the agents is introduced in the system, significantly reducing communication energy consumption. By employing the proposed control strategy, the Zeno behavior also can be avoided. Additionally, an obstacle avoidance control algorithm for the heterogeneous USV-AUV system based on improved artificial potential fields (IAPF) is designed, which helps in avoiding both static and dynamic obstacles. Compared to existing research, this algorithm reduces control input jitter, resulting in smoother obstacle avoidance paths. Through extensive simulation experiments and comparisons with other methods, effectiveness and superiority of the proposed algorithm is validated.

## Section 1: Introduction

AUV (Autonomous Underwater Vehicle) is an unmanned underwater vehicle capable of performing various tasks such as ocean exploration, underwater geological surveys, marine ecosystem monitoring, and underwater operations [1]. They can independently execute missions and operate efficiently and precisely in complex underwater environments through advanced navigation, sensing, and control technologies. With continuous technological advancements, AUVs are anticipated to assume a pivotal role in the future. bringing about further innovation and development. AUVs possess autonomy, flexibility, and adaptability, enabling them to carry out high-risk tasks in the ocean. How to fully utilize AUVs for search behaviors in complex and dynamic marine environments is a topic of significant research value. However, AUVs require positioning services supported by mother ships, which can limit their range and flexibility. Additionally, the cost of ship time is another factor that motivates researchers to develop new technologies to replace mother ships and reduce expenses. To overcome these limitations and improve the efficiency of AUVs, unmanned surface vehicles (USVs) are being explored as alternatives to mother ships, fulfilling the task of monitoring AUVs. As a result, researchers have begun studying multi-AUV systems [2]. By leveraging communication and cooperative abilities among AUVs through collaboration and task allocation, search efficiency and task coverage can be enhanced. Formation systems provide new approaches to the execution of maritime missions [3]. Consequently, in recent years, heterogeneous USV-AUV systems have gained attention in the field of marine engineering. Consensus poses as a fundamental concern in the collective behavior of multi-agent systems (MASs). Achieving consensus is crucial for coordination and cooperation among multiple agents, involving information exchange, decision-making, and dynamic adjustments [4]. In the context of consensus problems, all agents need to agree on joint actions to accomplish complex tasks [4].

In consensus mechanisms, the convergence rate serves as a crucial metric for ensuring consensus [5]. Current research on consensus primarily focuses on asymptotic convergence while assuming an infinite convergence time [6,7]. However, the requirements of many real systems are somehow unrealistic. Therefore, researchers have considered finite-time consensus control to better meet the time requirements of consensus in real systems and to enhance the system’s response speed and stability [8]. These criteria help to design control strategies that allow the system to reach a consistent state in a finite time [9]. Moreover, a robust finite-time event-triggered consensus (ETC) based on an integral sliding mode is proposed in [10]. Research in this area of finite-time consensus control problems is important for advancing the fields of distributed control, collaborative robotics, and intelligent networks. The finite-time ETC for heterogeneous linear MASs was addressed in [11]. The introduction of the concept of fixed time (prescribed finite time) stability was presented in [12]. It means that the system can achieve a steady state in a predefined finite time and is no longer influenced by the initial conditions. This stability concept is important for the control design of real systems to ensure that the system completes its task and meets the performance requirements in a specific time. Compared to the asymptotic consensus in [5–7], consensus in a fixed time can be achieved faster and with better disturbance rejection performance. In recent research, [13] focused on multi-agent systems (MASs) with nonlinear dynamics and uncertain interference in terms of robust fixed-time consensus. In addition, fixed-time consensus control methods of higher-order linear and nonlinear MASs, which are very important for solving problems in practical applications [14,15]. A distributed observer-based continuous consensus control algorithm was developed in [16], which enables continuous consensus for MASs.

However, input delay and fixed-time are not considered in the above results. Time delays often occur in MASs, which will appear as instability. Taking time delay into consideration is of utmost importance in control systems. The literature [17] pointed out the importance of time delay and provided related theoretical support. The consensus problem for linear and nonlinear MASs with input delay was presented in [18,19]. These studies aim to explore the nature of consensus and control strategies for MASs under input delay. In addition, the literature [20] with input delays for MASs, which is important to achieve fast and accurate consensus.

In the previously mentioned studies, frequent controller updates and communications are required, thus leading to a large amount of communication consumption. To address these drawbacks, several scholars have conducted related studies and proposed an ETC strategy to overcome these problems [21–23]. This strategy can intelligently select the event trigger timing and reduce the communication frequency. In [21], the ETC is proposed for first-order MASs. In [22], a decentralized ETC is presented for linear low-order MASs. In [23], ETC is implemented in discrete-time MASs. In the literature [5], an ETC framework for nonlinear leaderless multi-intelligent systems (MAS) was proposed, but the method could not avoid Zeno behavior. To address this problem, in the literature [24], an ETC strategy is proposed that aims to avoid Zeno behavior. The goal of this study is to improve the stability and performance of the system while guaranteeing consensus. Furthermore, in the literature [25], an adaptive ET method is utilized for resolving the problem of consensus.

In all the aforementioned theoretical studies on the formation of consensus control, the issue of environmental barriers has not been considered. In practical applications, the existence of environmental obstacles in heterogeneous USV-AUV formations cannot be ignored. To solve this problem, scholars have proposed various methods, including artificial potential field method (APF) [26], model predictive control (MPC) [27], online optimal control [28], and reinforcement learning (RL) [29]. For efficient distributed MASs navigation, a novel end-to-end framework to generate reactive collision avoidance policy is presented in [30]. The online optimal control and MPC require a lot of calculation, which will reduce the real-time performance when the formation size is large [31]. The widely employed APF is favored for its straightforward structure and minimal parameter design [32]. However, there are still some challenges in the existing research. For example, a large number of computations are required, in situations where the formation size is substantial, which can degrade the real-time performance [31]. In addition, APF, although widely used, was found in [33] that the target may not be reached when there are obstacles nearby (GNWON) and has jitter problems. Existing studies have addressed the formation obstacle avoidance problem to some extent, but their effects have rarely been considered. Therefore, further research needs to consider the environmental disturbance factors and design obstacle avoidance strategies that adapt to dynamic obstacles. In addition, the interactions and potential field effects among multiple agents in the formation need to be considered to achieve more stable and efficient formation navigation.

In addition to this, it is evident from the above studies that most of the research has been conducted on MASs, such as unmanned aerial vehicles, yet there has been very limited research on AUV systems. In the underwater environment, factors such as currents, submarine topography, and underwater obstacles are involved, all of which pose additional challenges for the navigation and control of AUVs. Even fewer studies have been conducted on heterogeneous USV-AUV MASs. Some literature has considered the issue of time delay of the system [34,35]. Therefore, the fixed-time ETC strategy in [24] for multi-agent is applied to the heterogeneous USV-AUV MASs in this paper. On the basis of [24], input delay and obstacle avoidance are considered. Besides, the control method proposed in this paper can be applied to control performance optimization. Fixed-time event-triggered consensus control strategy can achieve the desired formation in multi-agent systems with varying constraints and topology changes, and it can optimize performance by adjusting the time intervals for triggering events reasonably. However, it may not be the optimal choice for control performance optimization, fixed-time event-triggered consensus control is typically better suited for maintaining system stability and coordination. In summary, the main problems in the existing research on multi-AUV formation control are as follows:

1. A single AUV is inefficient and requires positioning services supported by the mother ship, with low flexibility and low operational range.
2. The convergence rate of multi-AUV formation is slow, and frequent control updates result in significant communication consumption.
3. There are input-delay and obstacles in actual multi AUV systems, but few existing research considered.

Driven by the above problems, this study investigates fixed-time ETC for obstacle avoidance in heterogeneous USV-AUV MASs with input-delay and uncertain disturbances. By designing and analyzing the control algorithm, we achieve obstacle avoidance and consensus tasks in complex environments. This approach reduces the communication overhead and computational load by utilizing a fixed-time event triggering mechanism, and effectively addresses the challenges posed by input delays and uncertain interference. Through simulation experiments, the proposed algorithm shows commendable performance in heterogeneous USV-AUV MASs, providing valuable insights for further research and practical applications. The primary contributions of this paper are outlined as follows:

1. An obstacle-avoiding heterogeneous USV-AUV MASs simulating the actual environment is considered. The mother ship that supports positioning services is designed as a USV to improve efficiency and reduce costs. The APF algorithm is improved to solve the obstacle avoidance problem.
2. A fixed-time ETC strategy to guarantee convergence speed and reduce communication energy consumption is proposed. For arbitrary initial states, the convergence time of heterogeneous MASs can be upper bounded by a positive constant.
3. The input delay is taken into account in the proposed heterogeneous USV-AUV system to improve the stability and accuracy of the system.

The subsequent sections of this paper are structured as follows. In Section 2, the preliminaries are provided, presenting the notations, algebraic graph theory, and background of the research. In Section 3, we develop a fixed-time ETC with obstacle avoidance for heterogeneous multi-agent systems. The design principles and algorithms of the method are described in detail. In Section 4, the simulation experiments are conducted to validate the proposed method’s performance and effectiveness in complex environments. Finally, the research content of this article as well as discusses potential directions for future research and application are summarized in Section 5.

## Section 2: Preliminaries

First, we briefly introduce the basic concepts and principles of notations and algebraic graph theory, including the definition and operation rules of notations, and graph structures and algebraic operations in algebraic graph theory. Then, we discuss the relevant background knowledge in detail. Finally, we explicitly present the problem we are trying to solve. By stating the problem in this way, we lay the foundation for the subsequent research work.

### Notations

In this paper, ‖⋅‖ denote the Euclidean norm. The 2-norm of state vector $\eta (t)={\left[\eta_{1}(t),\eta_{2}(t),\ldots ,\eta_{N}(t)\right]}^{T}$ is expressed by ‖*η*(*t*)‖_2_. *diag*(⋅) represents the diagonal Matrix. Let ℜ^*n*^ and ℜ^*n*×*n*^ represent the real space of *n*-dimensional column vectors and *n*×*n*-dimensional matrices, respectively.

### Algebraic graph theory

The shape of the heterogeneous USV-AUVs multi-agent system is represented by an undirected graph *G*(*ν*, *E*). An edge of *G* is manifested by *e*_*ij*_ = (*ν*_*i*_, *ν*_*j*_). The neighborhood set of *ν*_*i*_ is denoted by $N_{i}=\{j\in \nu :(\nu_{i},\nu_{j})\in E\}$. The adjacency matrix is *A*, *a*_*ij*_ is called the weight of link (*ν*_*i*_, *ν*_*j*_). If (*ν*_*i*_, *ν*_*j*_)∈*e*_*ij*_, *a*_*ij*_ = 1, otherwise *a*_*ij*_ = 0. *D* = *diag*[*d*_1_,…,*d*_*N*_] is the degree matrix, $d_{i}=\sum_{j=1,j\neq i}^{N}a_{ij}$. The Laplacian matrix *L* = *D*−*A* = [*l*_*ij*_]∈ℜ^*n*×*n*^.

### Background

**Fig 1:**
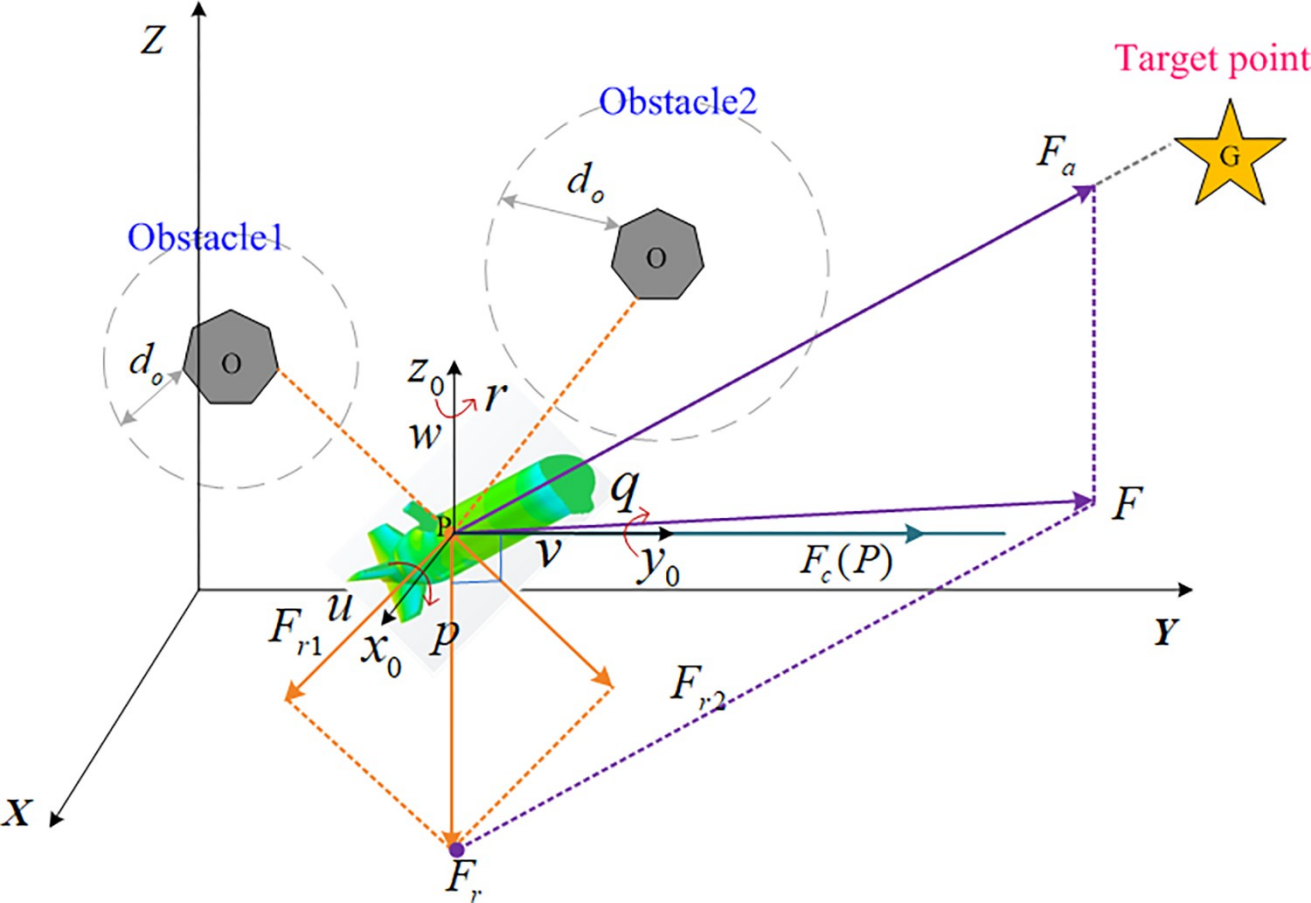
The schematic model of agent.

In order to provide a more comprehensive description of the motion of heterogeneous USV-AUV multi-agent system, the inertial coordinate (*X*−*Y*−*Z*) and body-fixed coordinate (*x*_0_−*y*_0_−*z*_0_) are established, as shown in Fig 1. This section presents the introduction to the kinematics and dynamics of both the USV and AUV.

- The Model of USV

On the USV model, the 3-DOF kinetics are as follows [36].

$$\begin{array}{l} \dot{\eta }(t)=J(\eta )v=f(t,\eta (t))\\ M\dot{v}=-C(v)v-D(v)v+\tau \end{array}$$ (1)

where **η** = [*x*,*y*,*ψ*]^*T*^, **v** = [*u*,*v*,*r*]^*T*^ represent the position and velocity vector. **τ** is the control input vector. The inertia matrix **M**∈*R*^3×3^, rotation matrix **J**∈*R*^3×3^, Coriolis-centripetal matrix **C**∈*R*^3×3^ and damping matrix **D**∈*R*^3×3^ are considered in horizontal plane and given as follows.

- The Model of AUV

In the AUV model, 6-DOF kinetics were used [37].

$$\begin{array}{l} \dot{\eta }(t)=J(\eta )v=f(t,\eta (t))\\ M\dot{v}=-C(v)v-D(v)v-g(\eta )+\tau \end{array}$$ (2)

where **η** = [*x*,*y*,*z*,*φ*,*θ*,*ψ*]^*T*^, **v** = [*u*,*v*,*w*,*p*,*q*,*r*]^*T*^ and **τ** are same as the previous. **J**(*η*)∈*R*^6×6^ is the rotation matrix, which is defined as:

$$J(\eta )=[\begin{array}{cc} J_{T}(\eta ) & 0_{3\times 3}\\ 0_{3\times 3} & J_{R}(\eta ) \end{array}],$$

where **J**_*T*_∈*R*^3×3^ and **J**_*R*_∈*R*^3×3^, **0**_3×3_∈*R*^3×3^ is the matrix of zeros.

There are the following definitions and lemmas.

*Definition 1[21]:* If there is a settling time *T*(*η*_0_)>0, and the origin of (1) is asymptotically stable, it is globally finite-time stable. The solution *η*(*t*,*η*_0_) can reach the equilibrium in *T*(*η*_0_). Besides, if ∃*T*_max_>0, *T*≤*T*_max_, then it is fixed-time stable.

*Lemma 1 [17]:* For graph *G*, the Laplace matrix *L* of the undirected graph possesses the following properties.

$$\eta^{T}L\eta =\frac{1}{2}\sum_{i=1}^{N}\sum_{j=1}^{N}a_{ij}{\left(\eta_{i}-\eta_{j}\right)}^{2}$$

where *η* = [*η*_1_,*η*_2_,…,*η*_*N*_]^*T*^. The eigenvalues of *L* are assumed label as 0,*λ*_2_,…,*λ*_*N*_, and 0≤*λ*_2_≤…≤*λ*_*N*_. Then, if **1**^*T*^*η* = 0, there is *η*^*T*^*Lη*≥*λ*_2_*η*^*T*^*η*.

*Lemma 2[38]:* If there is a Lyapunov function *W*(*t*), so that

$$\dot{W}(\eta (t))\leq -aW^{p}(\eta (t))-bW^{q}(\eta (t))$$

where *a*>0, *b*>0, *p*∈(0,1), and *q*>1. Consequently, the origin of the system (1) exhibits fixed-time stability, there is

$$T\leq T_{\max}≔\frac{1}{a(1-p)}+\frac{1}{b(q-1)}$$

where *T* is the settling time. In addition, if

$$\dot{W}(\eta (t))\leq -aW^{p}(\eta (t))-bW^{q}(\eta (t))+\vartheta ,\vartheta \in (0,\infty )$$

As a result, the trajectory of system (1) attains practical fixed-time stability.

*Lemma 3 [39]:* Let *θ*_1_,*θ*_2_,…,*θ*_*N*_≥0, then

$$\sum_{i=1}^{N}\theta_{i}^{p}\geq {\left(\sum_{i=1}^{N}\theta_{i}\right)}^{p},\;0<p\leq 1$$

$${\left(\sum_{i=1}^{N}\theta_{i}\right)}^{q}\geq \sum_{i=1}^{N}\theta_{i}^{q}\geq N^{1-q}{\left(\sum_{i=1}^{N}\theta_{i}\right)}^{q},\;1<q\leq \infty$$

- D. Problem Statement

Consider the heterogeneous USV-AUV MASs composed of *N* agents. The dynamics model of agent *i* expressed as follows.

$${\dot{\eta }}_{i}(t)=u_{i}(t-\tau_{i})+\omega_{i}(\eta_{i}(t),t)$$ (3)

where *η*_*i*_ is the state, *u*_*i*_ is the control input, *τ*_*i*_ is the input delay, and *ω*_*i*_(*i* = 1,2,…,*N*) is the uncertain disturbances (such as the wind, wave and current) of the heterogeneous USV-AUV system.

The fixed-time consensus for heterogeneous USV-AUV has

$$\underset{t\to T}{\lim}|\eta_{i}(t)-\eta_{j}(t)|=d_{ij}(t)$$ (4)

where *d*_*ij*_ is the desired pose between agent *i* and *j*. When *t*≥*T*, *η*_*i*_(*t*)−*η*_*j*_(*t*) = *d*_*ij*_(*t*).

*Assumption 1*: *ω*_*i*_(*η*_*i*_(*t*),*t*) is bounded by *ϖ*, that is

$$|\omega_{i}(\eta_{i}(t),t)|\leq \varpi .$$ (5)

## Section 3: The Fixed-time ETC with obstacle avoidance

Firstly, the traditional APF is improved, used as the obstacle avoidance planning algorithm of heterogeneous USV-AUV formation in this section. Then, a fixed-time ETC is presented.

### Improved artificial potential field

In the potential field, *F*_*a*_ is the attractive force of the target point, *F*_*r*_ is the repulsive force of the obstacle. *F* is the combined force on USV/AUV. To address the challenge posed by dynamic obstacles, we modify the repulsive potential field function. The heterogeneous USV-AUV formation is able to continuously avoid obstacles. With the improved repulsive potential field function *U*_*r*_, the formation is able to avoid dynamic obstacles more intelligently, improving the safety and efficiency of navigation. To avoid dynamic and static obstacles, we introduce an adaptive repulsion coefficient *k*_*r*_ based on the movement speed of dynamic obstacles. By redefining *U*_*r*_, this enables the USV/AUV to avoid obstacles more effectively. The *U*_*r*_ is:

$$U_{r}=\{\begin{array}{l} \frac{1}{2}k_{r}(\frac{d_{po}}{d_{o}^{2}}-\frac{2\ln d_{po}}{d_{o}}-\frac{1}{d_{po}}),\;0<d_{po}<d_{o}^{}\\ 0,\;d_{po}\geq d_{o}^{} \end{array}$$ (6)

The repulsive function is expressed as Eq (7).

$$F_{r}=-\nabla U_{rep}=\{\begin{array}{l} \frac{1}{2}k_{r}(-\frac{1}{d_{o}^{2}}+\frac{2}{d_{o}d_{po}}-\frac{1}{d_{po}^{2}}),\;0<d_{po}<d_{o}\\ 0,\;d_{po}>d_{o} \end{array}$$ (7)

where $k_{r}=\{\begin{array}{l} 1,\;v_{o}=0\\ 1+e^{v_{o}/\rho },\;v_{o}\neq 0 \end{array}$, *v*_*o*_ is the speed of the obstacle. The Euclidean distance between the obstacle point and the USV/AUV is represented by *d*_*po*_. The rounding of *d*_*po*_ is represented as *ρ* = [*d*_*po*_]. *d*_*o*_ is the range of obstacle rejection, i.e., the obstacle will no longer affect when the distance is greater than *d*_*o*_.

To address this problem of unreachable targets and the presence of obstacles (GNWON) in the conventional attractive potential field method, an attraction influence factor $d_{po}^{m}$ is introduced. In addition, to overcome the dilemma that agents are easily caught in local minima in the conventional APF, an auxiliary force consisting of an attractive force and a repulsive force is introduced. The schematic diagram is represented by Fig 1. The attraction potential function can be represented by Eq (8).

$$U_{a}=\{\begin{array}{l} \frac{1}{2}k_{a1}d_{pg}{}^{2}d_{po}^{m},\;d_{pg}<d_{r}\\ \frac{1}{2}k_{a2}d_{pg}{}^{2}d_{po}^{m},\;d_{pg}\geq d_{r} \end{array}$$ (8)

where *k*_*a*1_ and *k*_*a*2_ are the attraction coefficient. The attraction function is represented by the following.


$$F_{a}=-\nabla U_{a}=\{\begin{array}{l} -k_{a1}d_{pg}d_{po}^{m}\\ -\frac{mk_{a1}}{2}d_{pg}^{2}d_{po}^{m-1},\;d_{pg}<d_{r}\\ -k_{a2}d_{pg}d_{po}^{m}\\ -\frac{nk_{att2}}{2}d_{pg}^{2}d_{po}^{m-1},\;d_{pg}\geq d_{r} \end{array}$$ (9)

The auxiliary force is expressed by the following equation.

$$F_{c}=\{\begin{array}{l} \varepsilon (\|F_{r}\|+\|F_{a}\|)\frac{F_{a}F_{r}^{2}}{\|F_{a}F_{r}^{2}\|}\frac{F_{a}}{\|F_{a}\|},\;d_{po}\leq \gamma d_{o}\\ 0,\;d_{po}>\gamma d_{o} \end{array}$$ (10)

where *ε* is the gain coefficient and *γ* is the distance coefficient.

In addition, the range of influence of the forces between agents is considered. When *d*_*ij*_>*d*_*r*_, an attractive potential field exists, and this attraction can be enhanced by adjusting the degree of increase of the parameter *d*_*ij*_. As *d*_*ij*_ increases, the attractive force increases accordingly, prompting agents to come closer to each other. The gravitational effect of this potential field helps to avoid collisions between agents. The potential field function of the attractive force is as follows.


$$U_{aij}=\sum_{j=1,j\neq i}^{N}a_{ij}\frac{1}{2}k_{aij}d_{ij}^{2}$$ (11)

The attractive function between agents is shown below.


$$F_{aij}=-\nabla U_{aij}=-\sum_{j=1,j\neq i}^{N}a_{ij}k_{aij}d_{ij}$$ (12)

When *d*_*ij*_<*d*_*r*_, the improved repulsive potential field function *U*_*rij*_ can be expressed by the following equation.


$$U_{rij}=\sum_{j=1,j\neq i}^{N}a_{ij}\frac{1}{2}k_{rij}{\left(\frac{1}{d_{ij}}-\frac{1}{d_{r}}\right)}^{2}.$$ (13)

The exclusivity between agents can be mathematically represented by the following equation.

$$F_{rij}=-\nabla U_{rij}=\sum_{j=1,j\neq i}^{N}a_{ij}\frac{k_{rij}}{d_{ij}^{2}}(\frac{1}{d_{ij}}-\frac{1}{d_{r}})$$ (14)

where *k*_*aij*_ = 1+*e*^*λ*^ and *k*_*rij*_ = 1+*e*^−*λ*^. The resultant forces *F* of USV/AUV is:

$$F=F_{a}+F_{r}+F_{c}+F_{aij}+F_{rij}.$$ (15)

As the result, the maneuvering speed difference is:

$$v_{i}=\frac{F_{i}(p)}{m_{i}}\delta_{t}$$ (16)

where *m*_*i*_ is the mass, *δ*_*t*_ is denoted as the simulation step length.

### Fixed-time ETC

The ETC of agent *i* is shown as (17).

$$u_{i}(t)=-\alpha \chi_{i}^{\mu }(t_{ki}^{i})-\bar{k}sign(\chi_{i}(t_{ki}^{i})),\;t\in [t_{ki}^{i},t_{ki+1}^{i})$$ (17)

where, *sign*(⋅) is the sign function, *χ*_*i*_(*t*) is

$$\chi_{i}(t)=\sum_{j=1}^{N}a_{ij}(\delta_{i}(t)-\delta_{j}(t))$$ (18)

$$\delta_{i}(t)=\eta_{i}(t)+\int_{t-\tau_{i}}^{t}u_{i}(T)dT$$ (19)

where *α* and $\bar{k}$ are:

$$\begin{array}{l} \alpha =(b{\left(2\lambda_{2}\right)}^{-\frac{\mu +1}{2}}N^{\frac{\mu -1}{2}}){\left(1-\zeta \right)}^{-1}\\ \bar{k}=(a{\left(2\lambda_{2}\right)}^{-\frac{1}{2}}+\varpi ){\left(1-\zeta \right)}^{-1} \end{array}$$ (20)

where, *λ*_2_ is the second smallest eigenvalue of *L*, *ζ*∈(0.1) is the triggering parameter. $t_{ki}^{i}$ is the latest event time for agent *i*.

The measurement error of each agent is designed as:

$$e_{i}(t)=\alpha \chi_{i}^{\mu }(t_{ki}^{i})+\bar{k}sign(\chi_{i}(t_{ki}^{i}))-\alpha \chi_{i}^{\mu }(t)-\bar{k}sign(\chi_{i}^{}(t)).$$ (21)

The ET function is considered as:

$$\varphi_{i}(t)=|e_{i}(t)|-\zeta (\alpha |\chi_{i}^{\mu }(t)|+\bar{k}).$$ (22)

Event is triggered when *φ*_*i*_(*t*)≥0, the controller of each agent is updated at its individual event time $t_{0}^{i},t_{1}^{i},\ldots$.

*Theorem 1*: The consensus problem of the heterogeneous USV-AUV MASs (3) with input delay and uncertain disturbances can be addressed by the control input (17).

*Proof*: Differentiating *δ*_*i*_(*t*) against *t*, according to Newton-Leibniz formula, the following equation can be obtained.

$$\begin{array}{l} {\dot{\delta }}_{i}(t)=u_{i}(t)+\omega_{i}(\eta_{i}(t),t)\\ \;=-\alpha \chi_{i}^{\mu }(t_{k}^{i})-\bar{k}sign(\chi_{i}^{}(t_{k}^{i}))+\omega_{i}(\eta_{i}(t),t) \end{array}$$ (23)

where $\delta (t)={\left[\delta_{1}(t),\delta_{2}(t),\ldots ,\delta_{N}(t)\right]}^{T}$, ${\dot{\delta }}_{i}(t)$ is discontinuous. Then, the set-valued Lie derivative and the concept of Filippov solutions should be applied [17]. The following Lyapunov function is constructed.


$$V(t)=\frac{1}{2}\delta^{T}(t)L\delta (t)$$ (24)

According to Lemma 1, we get

$$\lambda_{2}\delta^{T}(t)L\delta (t)\leq \sum_{i=1}^{N}\chi_{i}^{2}(t)\leq \lambda_{N}\delta^{T}(t)L\delta (t)$$ (25)

where the largest eigenvalue of *L* is denoted as *λ*_*N*_. According to Lemma 1 and Lemma3, the derivative of *V*(*t*) can be get as follows.


$$\begin{array}{l} \dot{V}(t)=\frac{1}{2}(\delta^{T}(t)L\dot{\delta }(t)+{\dot{\delta }}^{T}(t)L\delta (t))=\delta^{T}(t)L\dot{\delta }(t)\\ =\sum_{i=1}^{N}\sum_{j=1}^{N}a_{ij}(\delta_{i}(t)-\delta_{j}(t)){\dot{\delta }}_{i}(t)=\sum_{i=1}^{N}\chi_{i}(t)(u_{i}(t)+\omega_{i}(\eta_{i}(t),t))\\ =-\sum_{i=1}^{N}\chi_{i}(t)(e_{i}(t)+\alpha \chi_{i}^{\mu }(t)+\bar{k}sign(\chi_{i}(t))-\omega_{i}(\eta_{i}(t),t)\\ \leq \sum_{i=1}^{N}\left|\chi_{i}^{}(t)\right||e_{i}(t)|-\alpha \sum_{i=1}^{N}\chi_{i}^{\mu +1}(t)-\bar{k}\sum_{i=1}^{N}\left|\chi_{i}(t\right)|+\omega \sum_{i=1}^{N}\left|\chi_{i}(t\right)|\\ \leq \sum_{i=1}^{N}\left|\chi_{i}^{}(t)\right|(\zeta \alpha |\chi_{i}^{\mu }(t)|+\zeta \bar{k})-\alpha \sum_{i=1}^{N}\chi_{i}^{\mu +1}-\bar{k}\sum_{i=1}^{N}\left|\chi_{i}(t\right)|+\omega \sum_{i=1}^{N}\left|\chi_{i}(t\right)|\\ \leq -\alpha (1-\zeta )\sum_{i=1}^{N}\chi_{i}^{\mu +1}(t)-(\bar{k}(1-\zeta )-\omega )\sum_{i=1}^{N}\left|\chi_{i}(t\right)|\\ \leq -\alpha (1-\zeta )N^{\frac{1-\mu }{2}}{\left(\sum_{i=1}^{N}\chi_{i}^{2}(t)\right)}^{\frac{\mu +1}{2}}-(\bar{k}(1-\zeta )-\omega ){\left(\sum_{i=1}^{N}\chi_{i}^{2}(t)\right)}^{\frac{1}{2}}\\ \leq -\alpha (1-\zeta )N^{\frac{1-\mu }{2}}{\left(2\lambda_{2}V(t)\right)}^{\frac{\mu +1}{2}}-(\bar{k}(1-\zeta )-\omega ){\left(2\lambda_{2}V(t)\right)}^{\frac{1}{2}}\\ \leq -aV^{\frac{1}{2}}(t)-bV^{\frac{\mu +1}{2}}(t) \end{array}$$ (26)

The Lemma 2 is used, we have

$$\underset{t\to T(\delta )}{\lim}V(t)=0$$ (27)

$$T(\delta )\leq T_{\max}=\frac{2}{a}+\frac{2}{b(\mu -1)}$$ (28)

When $t=T(\delta )\leq \frac{2}{a}+\frac{2}{b(\mu -1)}$, implying that *u*_*i*_(*t*) will be zero. Besides, when $t\leq \frac{2}{a}+\frac{2}{b(\mu -1)}+\tau_{i}$, $\int_{t-\tau_{i}}^{t}u_{i}(T)dT$ will be zero. Consequently, $\underset{t\to T(\delta )}{\lim}\delta (t)=\eta (t)$ can be achieved when *t* = *T*(*η*)≤*T*_max_+max(*τ*_*i*_). And, *T*(*η*) is bounded as $T(\eta )\leq \frac{2}{a}+\frac{2}{b(\mu -1)}+\max(\tau_{i})$.

Therefore, a consensus can be reached and the proof is finished. To avoid high frequency switching, we introduce a saturation function with the controller of agent *i* as:

$$u_{i}(t)=-\alpha \chi_{i}^{\mu }(t_{ki}^{i})-\bar{k}sat(\chi_{i}(t_{ki}^{i})),\;t\in [t_{ki}^{i},t_{ki+1}^{i}).$$ (29)

$$sat(\eta )=\{\begin{array}{l} \eta /\Delta ,\;|\eta |\leq \Delta \\ sign(\eta ),\;|\eta |>\Delta \end{array}$$ (30)

where, Δ is a very small positive constant. The measurement error of agent *i* can be expressed as follows:

$${\tilde{e}}_{i}(t)=\alpha \chi_{i}^{\mu }(t_{ki}^{i})+\bar{k}sat(\chi_{i}(t_{ki}^{i}))-\alpha \chi_{i}^{\mu }(t)-\bar{k}sat(\chi_{i}^{}(t))$$ (31)

The ET function is

$$\phi_{i}(t)=|{\tilde{e}}_{i}(t)|-\zeta (\alpha |\chi_{i}^{\mu }(t)|+\bar{k})$$ (32)

When *ϕ*_*i*_(*t*)≥0, the event is triggered. Guided by the Eq (29) and Eq (32), we successfully achieve a practical fixed-time consensus for heterogeneous USV-AUV MASs. Based on the previously mentioned assumptions of the Lyapunov candidate function, similar to Theorem 1, the proof procedure is omitted here. With this work, we have confirmed the robustness and reliability of the algorithm in the face of delays and disturbances, providing a solid foundation for practical applications. This has important implications for further research and development of control methods for heterogeneous USV-AUV MASs.

*Theorem 2*: Under Eq (29) and Eq (32) proposed in this paper, for the heterogeneous USV-AUV MASs, no Zeno behavior occurs.

*Proof*: (25) and (26) are considered, there is

$$\sum_{i=1}^{N}\chi_{i}^{2}(t)\leq 2\lambda_{N}V(t)\leq 2\lambda_{N}V(0)$$

with

$$V(0)=\frac{1}{2}\delta^{T}(0)L\delta (0).$$

When *χ*_*i*_(*t*)<Δ, according to (31), there is

$$\begin{array}{l} D^{+}|{\tilde{e}}_{i}(t)|\leq |{\dot{\tilde{e}}}_{i}(t)|=|(-\alpha \chi_{i}^{\mu }(t)-\bar{k}sat(\chi_{i}(t)))'|\\ \;\leq (|\alpha \mu \chi_{i}^{\mu -1}(t)|+\frac{\bar{k}}{\Delta })|{\dot{\chi }}_{i}(t)|\\ \;\leq {\left(\alpha \mu (2\lambda_{N}V(0)\right)}^{\frac{\mu -1}{2}}+\frac{\bar{k}}{\Delta })|\sum_{j=1}^{N}a_{ij}({\dot{\delta }}_{i}(t)-{\dot{\delta }}_{j}(t))|\\ \;\leq (\rho_{1}+\frac{\bar{k}}{\Delta })|\sum_{j=1}^{N}a_{ij}(u_{i}(t)+\omega_{i}(\eta_{i},t)-u_{j}(t)-\omega_{j}(\eta_{i},t))|\\ \;\leq (\rho_{1}+\frac{\bar{k}}{\Delta })|\sum_{j=1}^{N}a_{ij}(u_{i}(t)-u_{j}(t)+2l_{ii}\varpi |\\ \;\leq (\rho_{1}+\frac{\bar{k}}{\Delta })(|\sum_{j=1}^{N}l_{ij}u_{j}(t_{k_{j}}^{j})|+2l_{ii}\varpi )\\ \;\leq (\rho_{1}+\frac{\bar{k}}{\Delta })\rho_{2}(t_{k_{j}}^{j}) \end{array}$$ (33)

where,

$$\begin{array}{l} \rho_{1}=\alpha \mu {\left(2\lambda_{N}V(0)\right)}^{\frac{\mu -1}{2}}\\ \rho_{2}(t_{k_{j}}^{j})=|\sum_{j=1}^{N}l_{ij}(\alpha (\chi_{j}(t_{k_{j}}^{j}))\mu +\bar{k}sat(\chi_{j}(t_{k_{j}}^{j})))|+2l_{ii}\varpi \end{array}$$ (34)

Where $t_{k_{j}}^{j}$ is the latest event time instant. We get $\left|{\dot{\tilde{e}}}_{i}(t)|\leq \rho_{1}\rho_{2}(t_{k_{j}}^{j}\right)$, when *χ*_*i*_(*t*)>Δ. Considering (33), and ${\tilde{e}}_{i}(t_{k}^{i})=0$, we have

$$|{\tilde{e}}_{i}(t)|\leq \int_{t_{k}^{i}}^{t}|\dot{\tilde{e}}(s)|ds\leq \int_{t_{k}^{i}}^{t}(\rho_{1}+\frac{\bar{k}}{\Delta })\rho_{2}(t_{k_{j}}^{j})ds.$$ (35)

The next event of agent *i* will not be triggered before *ϕ*_*i*_(*t*) = 0 or $|{\tilde{e}}_{i}(t)|=\zeta \alpha |\chi_{i}^{\mu }(t)|+\zeta \bar{k}$, according to the triggering condition *ϕ*_*i*_≥0. Considering (35), we get

$$\begin{array}{l} |{\tilde{e}}_{i}(t_{k+1}^{i})|=\zeta \alpha |\chi_{i}^{\mu }(t_{k+1}^{i})|+\zeta \bar{k}\\ \;\leq \int_{t_{k}^{i}}^{t_{k+1}^{i}}(\rho_{1}+\frac{\bar{k}}{\Delta })\rho_{2}(t_{k_{j}}^{j})ds\\ \;\leq \int_{t_{k}^{i}}^{t_{k+1}^{i}}(\rho_{1}+\frac{\bar{k}}{\Delta })\rho_{2}(0)ds \end{array}$$ (36)

According to Eq (34) and Eq (36), it is easy to know that

$$\begin{array}{l} t_{k+1}^{i}-t_{k}^{1}\geq \frac{\zeta \bar{k}}{\left(\rho_{1}+\frac{\bar{k}}{\Delta })\rho_{2}(0\right)}\\ \rho_{2}(0)=\sum_{j=1}^{N}l_{ij}{\left(\alpha (2\lambda_{N}V(0)\right)}^{\frac{\mu }{2}}+\bar{k})+2l_{ii}\varpi \end{array}$$ (37)

implying that no Zeno behavior occurs. The proof has been completed.

## Section 4: Simulation results and discussion

Several simulations are performed to validate the effectiveness of the heterogeneous USV-AUV MASs algorithm under input delays and uncertain interference conditions in this section. These simulations are conducted to demonstrate the feasibility and stability of the proposed algorithm in real-world applications, and the results show that the algorithm can successfully cope with the challenges posed by latency and interference and achieve good performance. These validations provide a deeper understanding of the reliability and applicability of the algorithm and provide a basis for further research and applications.

### Simulation on the fixed-time ETC

**Fig 2:**
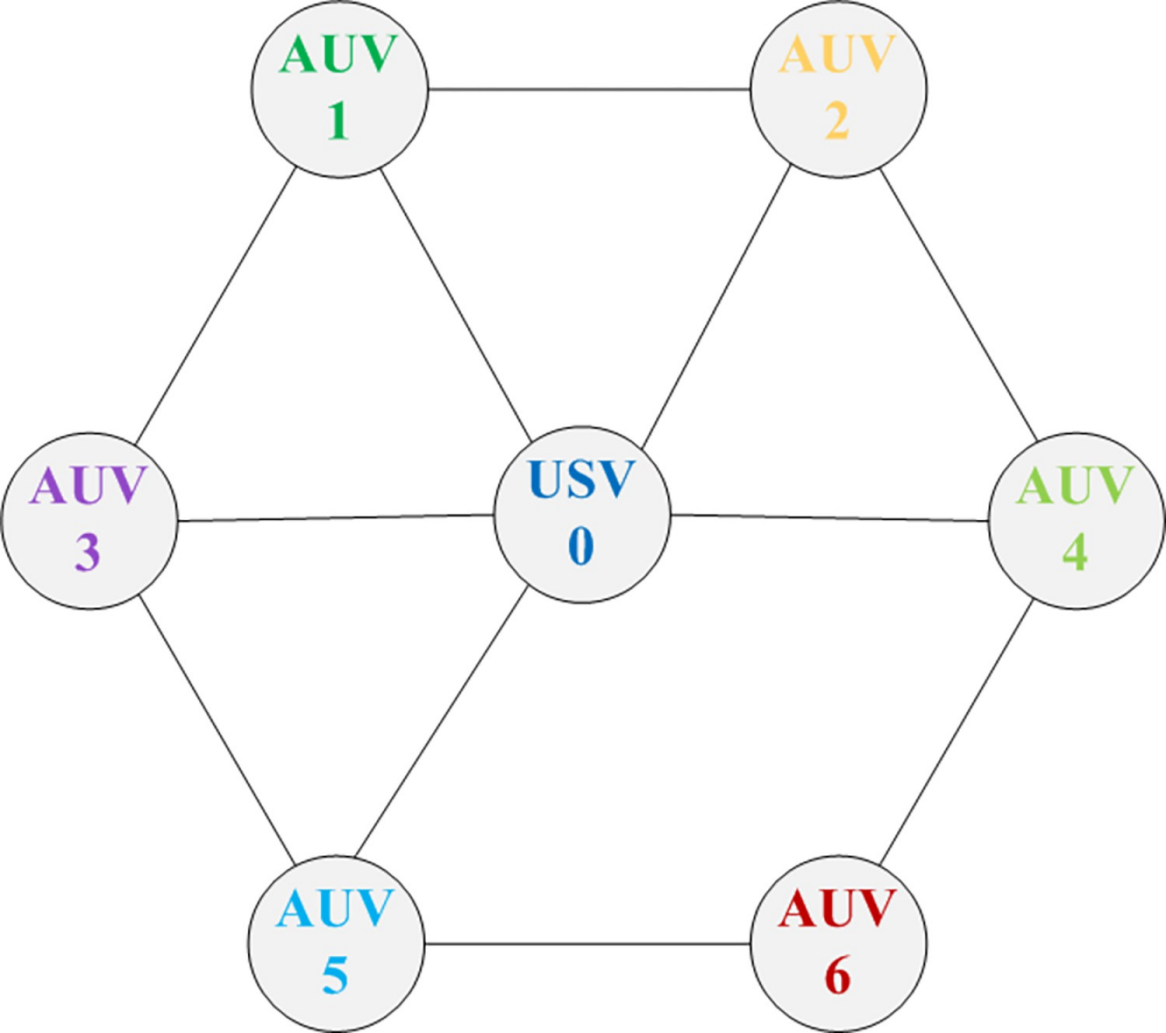
The communication graph.

Firstly, considering the heterogeneous USV-AUV MASs consisted of seven agents, the agent 0 is set as USV, and the other six agents are set as AUVs. The communication network topology is depicted in the Fig 2. The dynamics of agent *i* can be described by

$${\dot{\eta }}_{i}(t)=u_{i}(t-\tau_{i})+\omega_{i}(\eta_{i}(t),t)(i=1,2,3).$$ (38)

where *ϖ* = 0.2 in Assumption 1, $\omega_{i}(\eta_{i}(t),t)=0.2\cos(\eta_{i}(t))$, *τ*_0_ =*τ*_1_ =*τ*_2_ =*τ*_3_ =*τ*_4_ =*τ*_5_ =*τ*_6_ =0.03, *τ*_0_ represents the time delay of USV, *τ*_1_, *τ*_2_, *τ*_3_, *τ*_4_, *τ*_5_, *τ*_6_ represent the time delay of each AUV.

According to the undirected graph, the Laplacian matrix is as (39). Then, we get *λ*_2_ = 1.455, *λ*_*N*_ = 6.154. The parameters in the event-triggered consensus controller (17) are set as *a* = 0.2, *b* = 0.5, *μ* = 7/5, *ζ* = 0.9. Hence, *α* = 1.729, $\bar{k}=3.172$. On the basis of Theorem 1, *T*_max_ = 20.


$$L=[\begin{array}{ccccccc} 5 & -1 & -1 & -1 & -1 & -1 & 0\\ -1 & 3 & -1 & -1 & 0 & 0 & 0\\ -1 & -1 & 3 & 0 & -1 & 0 & 0\\ -1 & -1 & 0 & 3 & 0 & -1 & 0\\ -1 & 0 & -1 & 0 & 3 & 0 & -1\\ -1 & 0 & 0 & -1 & 0 & 3 & -1\\ 0 & 0 & 0 & 0 & -1 & -1 & 2 \end{array}]$$ (39)

The model parameters of USV are:

M=diag{2.583.383.38},

D=diag{1.51.850.2}.

The model parameters of AUV are:

M=diag{1.51.851.85},

D=diag{1.51.850.2}.

**Fig 3:**
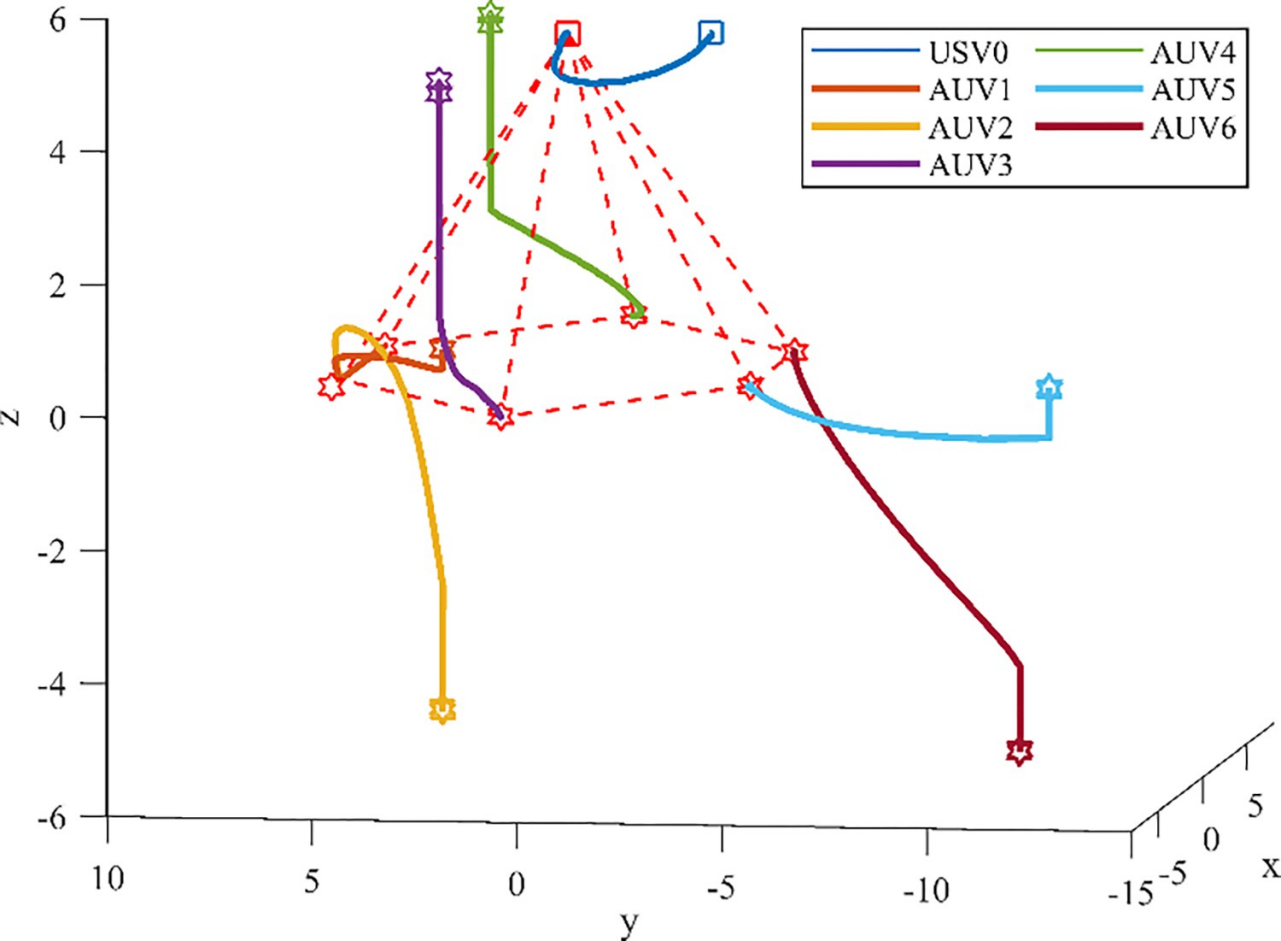
The process of formation generation for heterogeneous USV-AUV MASs.

**Fig 4:**
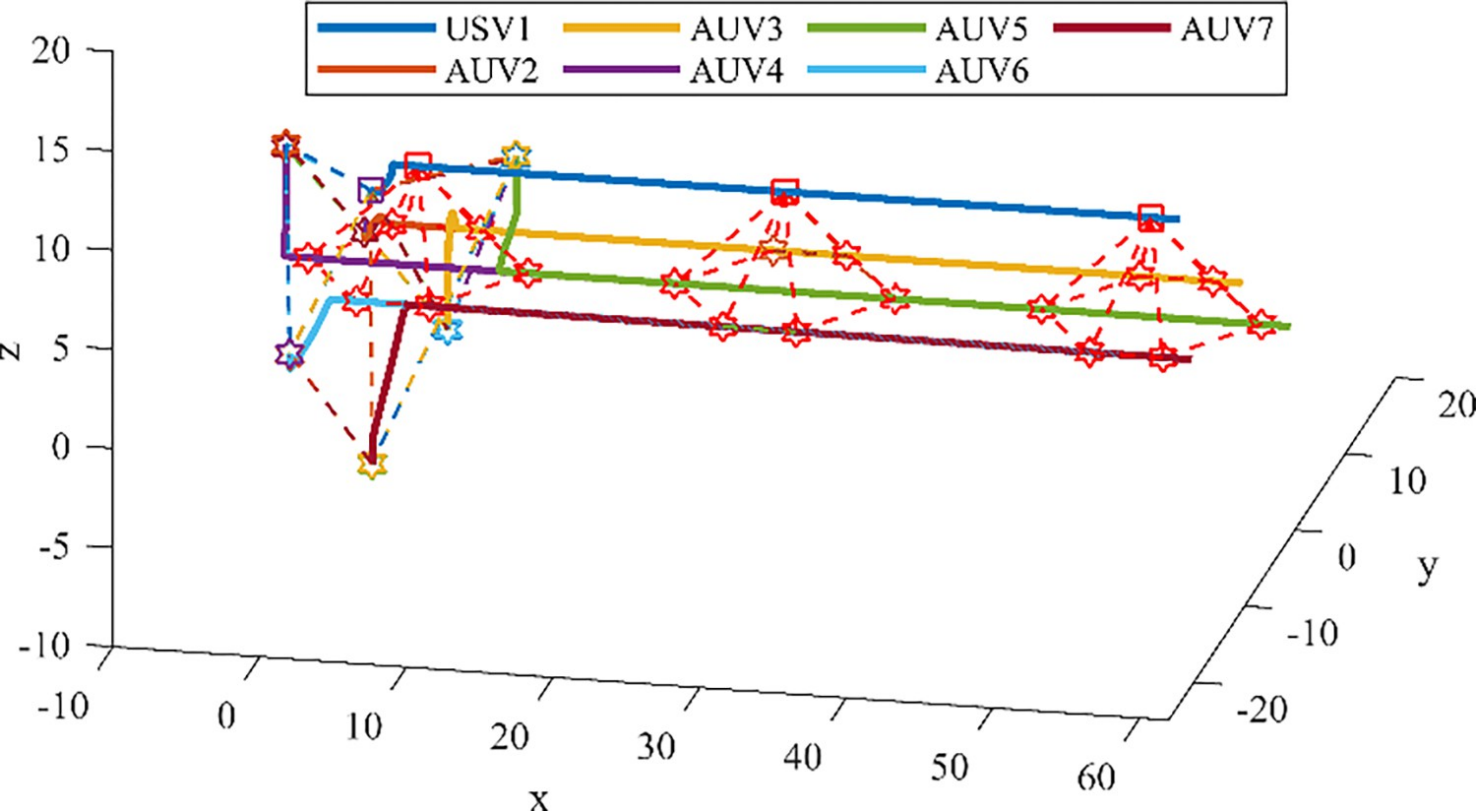
The formation path of the heterogeneous USV-AUV MASs with input delay is considered.

**Fig 5:**
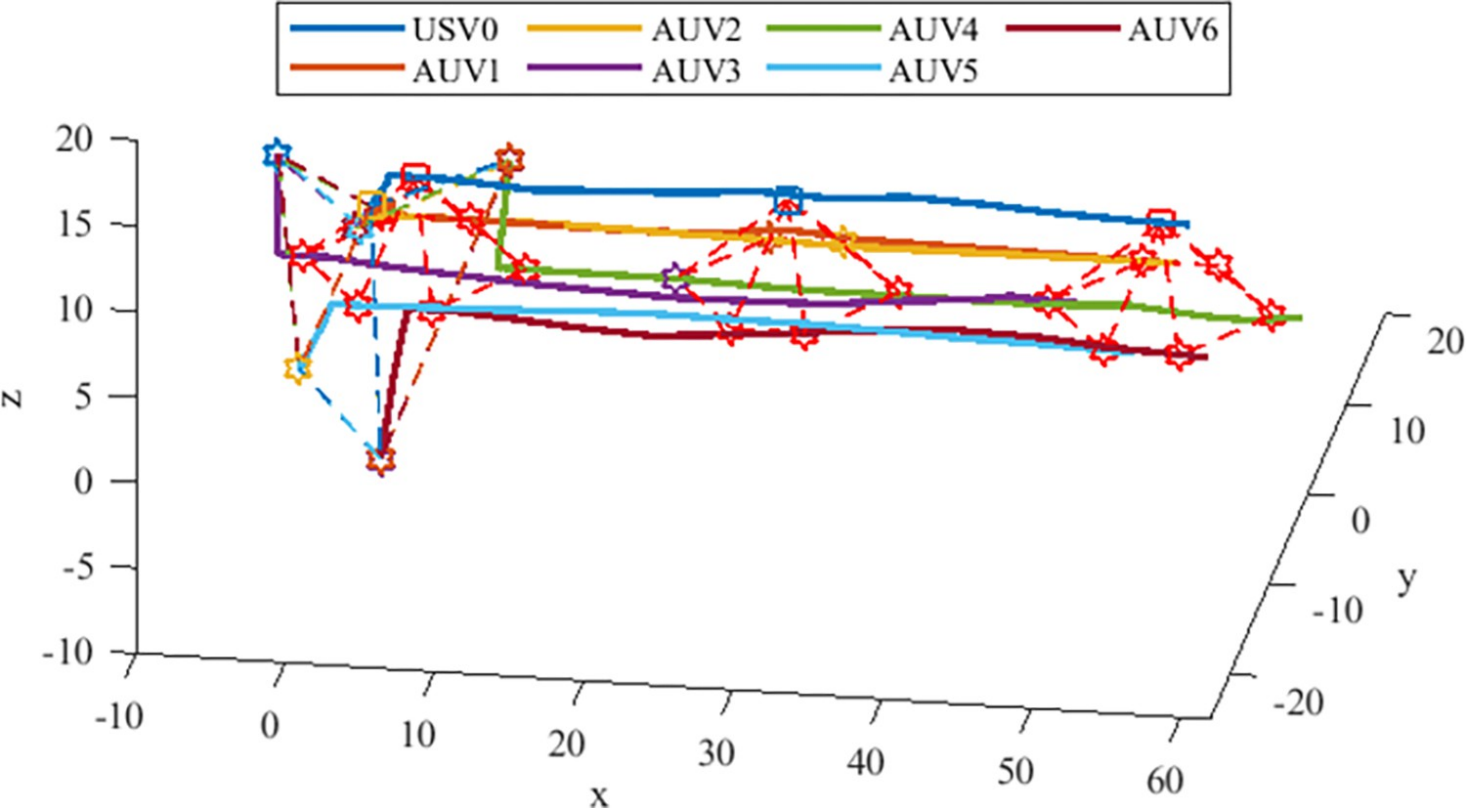
The formation path of the heterogeneous USV-AUV MASs when input delay is not considered.

The initial states of the seven agents are presented in the Table 1. The process of formation generation for heterogeneous MASs is shown in Fig 3. The formation path of the heterogeneous USV-AUV MASs is shown in the Fig 4. It can be easily known that the heterogeneous USV-AUV MASs can generate and maintain the desired formation under the control of the algorithm proposed in this paper from Figs 3 and 4. However, in the system with time delay, if delay is not considered, the system will be unstable, as shown in Fig 5.

**Table 1:** The state of the agents.

	USV0	AUV1	AUV2	AUV3	AUV4	AUV5	AUV6
**x**	0.5	-2	3	-8	7.5	-3	2.5
**y**	-3	3	5	2	4	-12	-10
**z**	5	0.5	-5	5	4.5	1	-6

**Fig 6:**
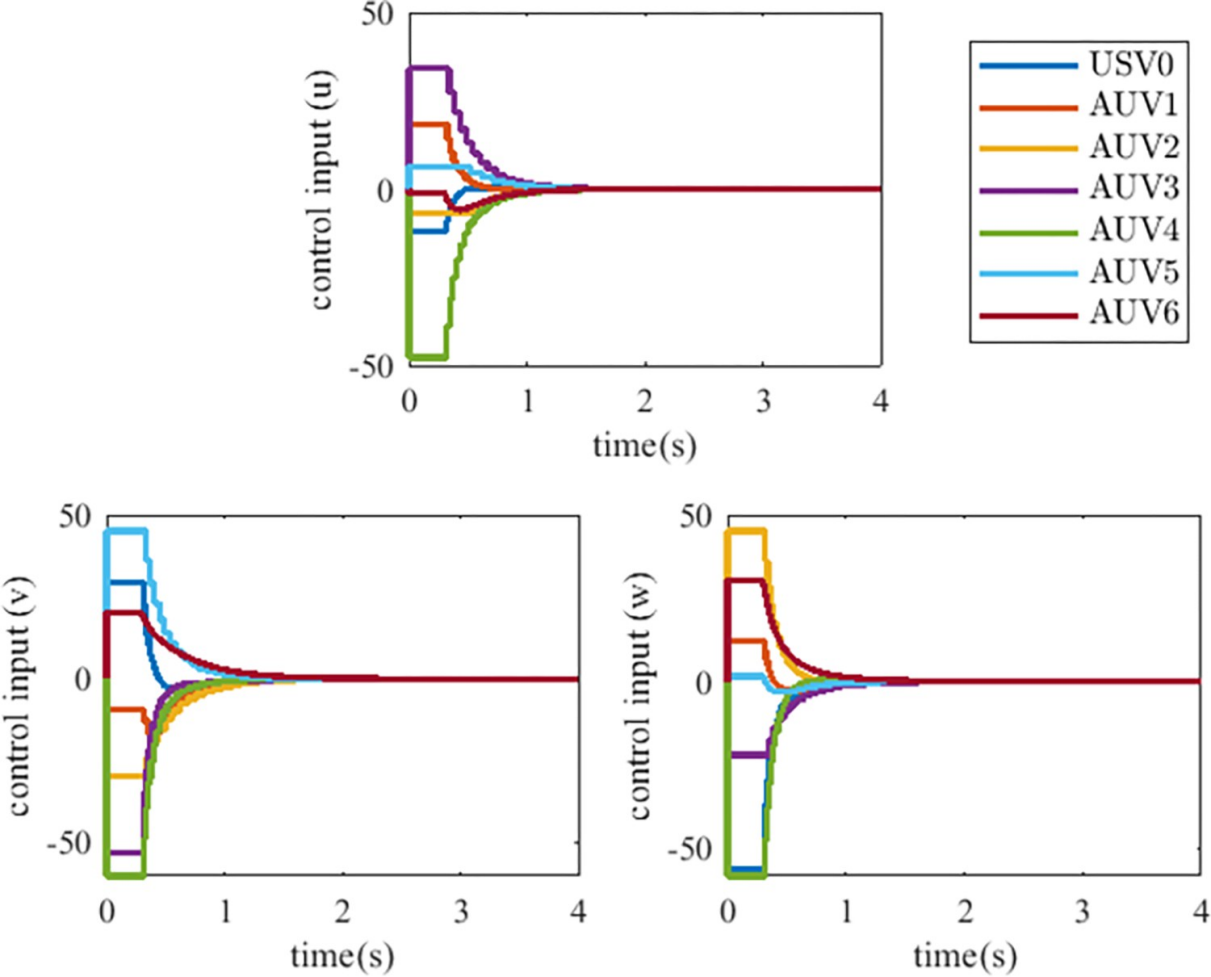
Control inputs of heterogeneous MASs with saturation function controller (29).

**Fig 7:**
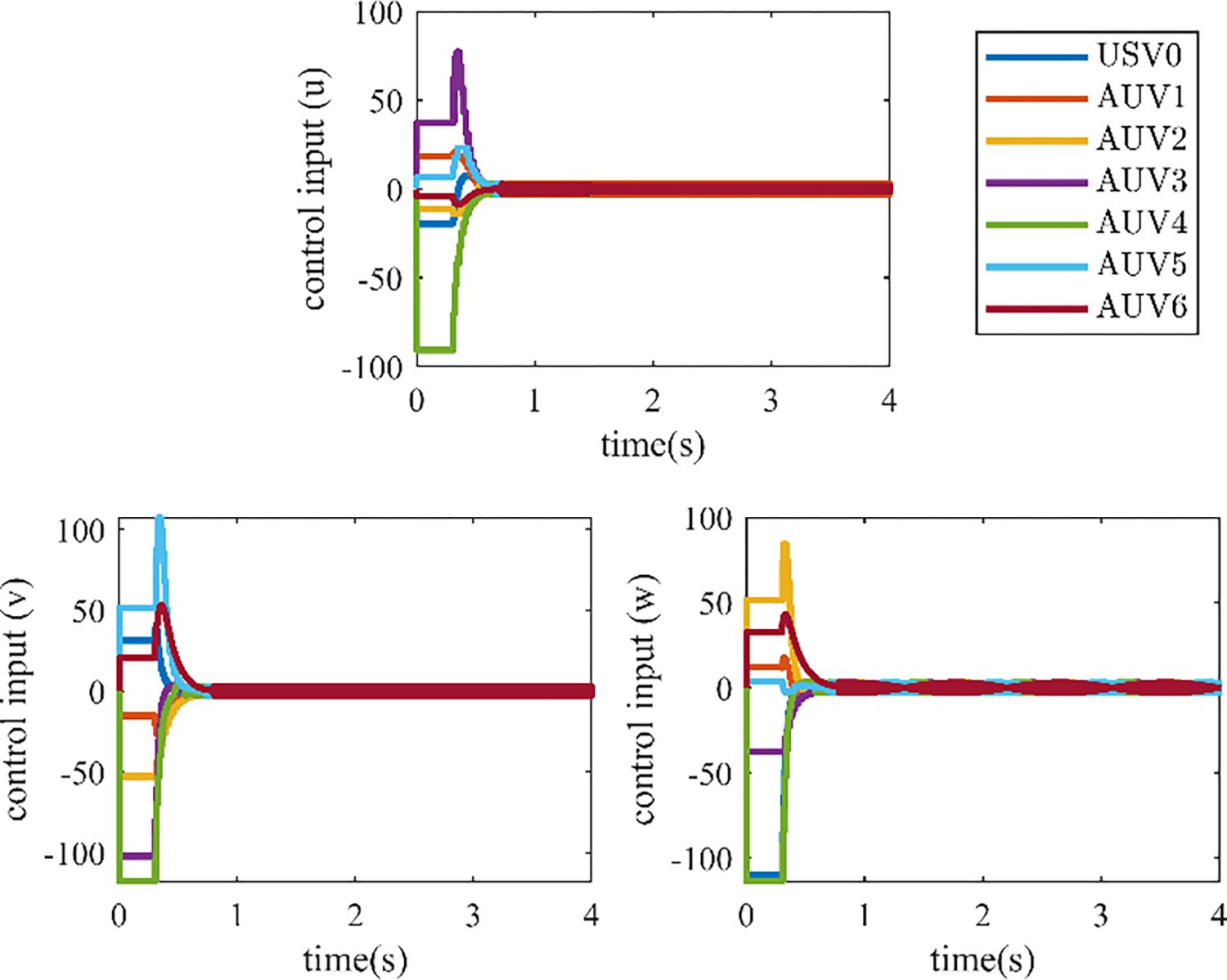
Control inputs of heterogeneous MASs with sign function controller (17).

**Fig 8:**
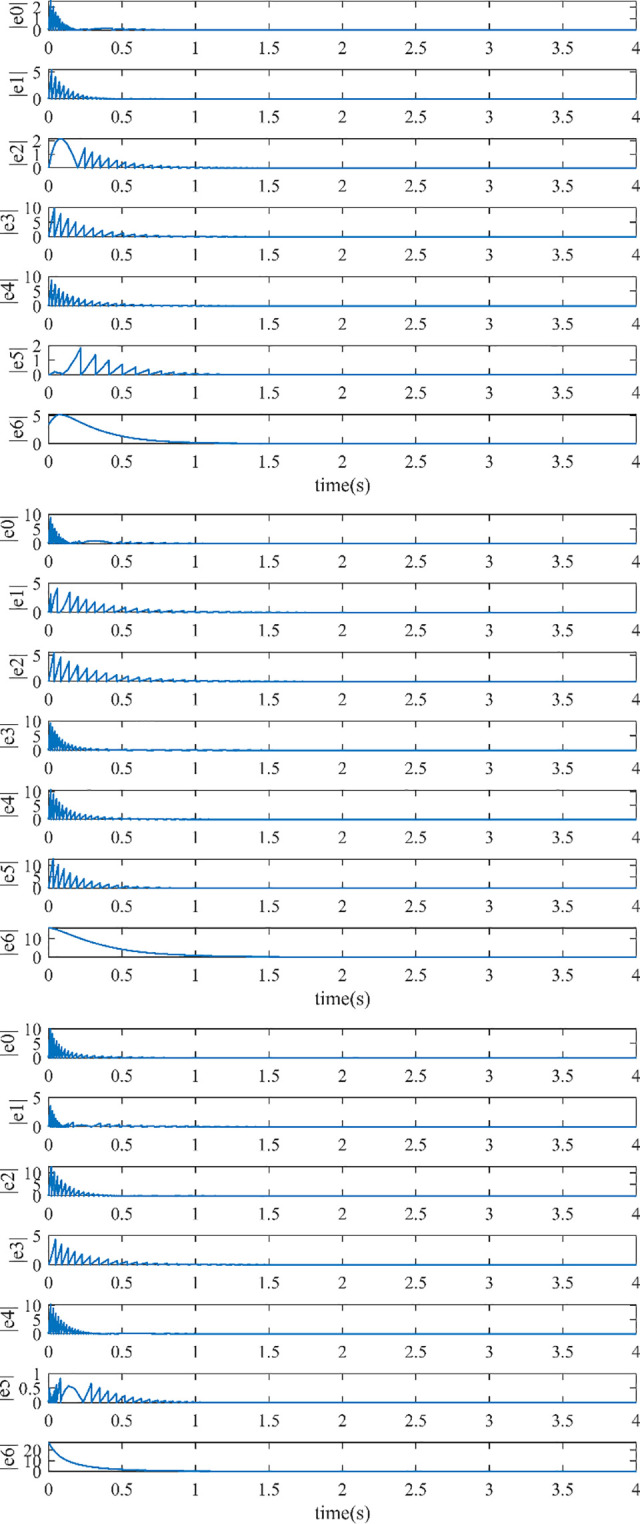
(a). The Measurement Error with Saturation Function Controller (29) in x-direction. (b). The Measurement Error with Saturation Function Controller (29) in y-direction. (c). The Measurement Error with Saturation Function Controller (29) in z-direction.

**Fig 9:**
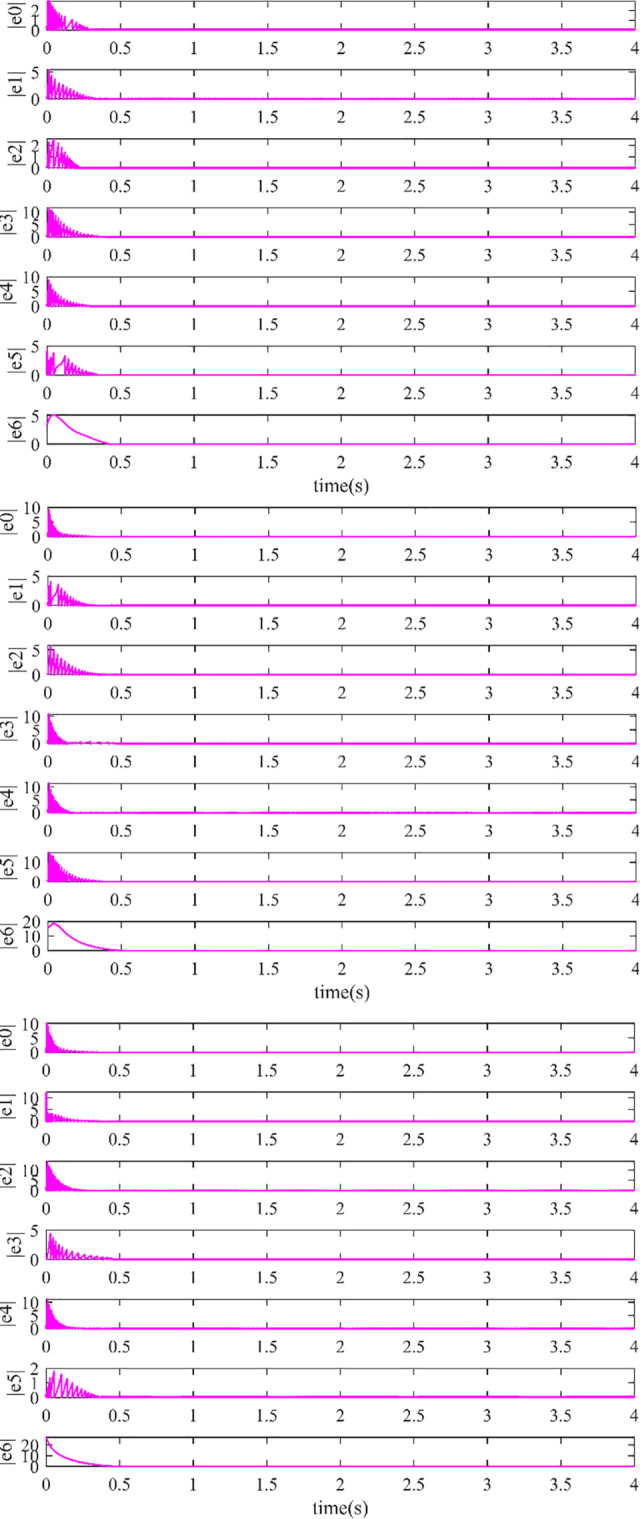
(a). The Measurement Error with Sign Function Controller (17) in x-direction. (b). The Measurement Error with Sign Function Controller (17) in y-direction. (c). The Measurement Error with Sign Function Controller (17) in z-direction.

**Fig 10:**
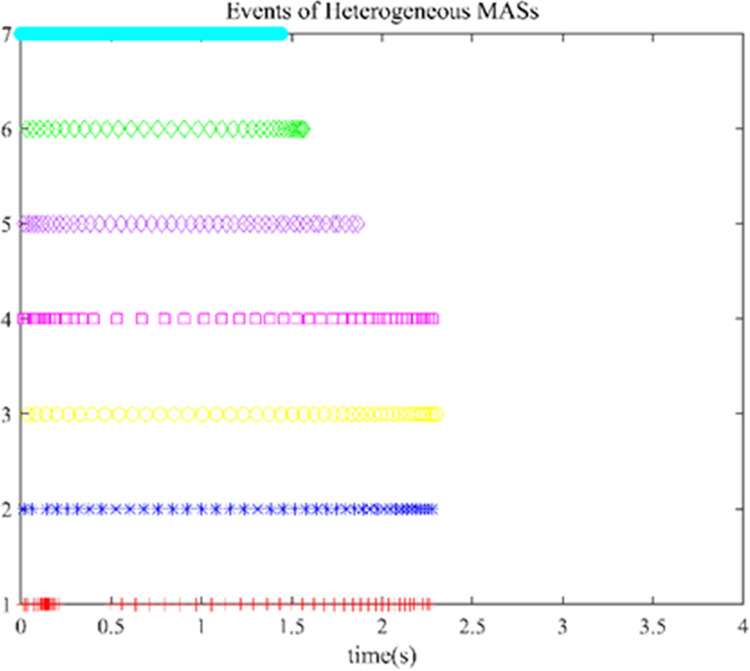
Event time instants of heterogeneous with saturation function controller (29).

**Fig 11:**
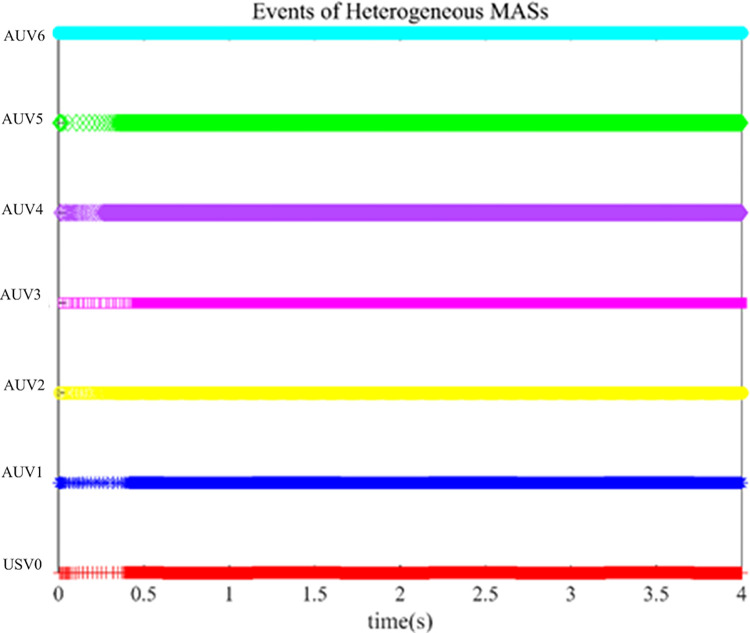
Event time instants of heterogeneous with sign function controller (17).

The controller (17) with the sign function is replaced by the controller (29) with saturation function thus reducing the chattering. Their control inputs are shown in Figs 6 and 7. The fluttering of the measurement error is demonstrated in Figs 8 and 9. The triggering instants of the heterogeneous MASs under the control of saturation function controller and sign function controller are illustrated in the Figs 10 and 11, respectively. The trigger times of each agent are displayed in Table 2. The event-triggered adopted in this paper only triggered when the state meets a certain condition, which greatly reduces the communication energy consumption. According to Figs 8 and 9, under the control of the sign function controller, the number of triggers is significantly more than that of the sat function controller. Obviously, the performance of sat function controller proposed in this paper is better than that of sign function controller.

**Table 2:** The Times of triggered for each agent.

Agent	Time-triggered	Event-triggered with sign controller (17)	Event-triggered with sat controller (29)
**USV0**	4000, 4000, 4000	1875,1812,1824	47,56,46
**AUV1**	4000, 4000, 4000	1849,1841,1815	57,42,52
**AUV2**	4000, 4000, 4000	1903,1815,1876	37,44,61
**AUV3**	4000, 4000, 4000	1817,1782,1781	33,45,42
**AUV4**	4000, 4000, 4000	1861,1901,1885	45,46,52
**AUV5**	4000, 4000, 4000	1842,1810,1839	26,34,45
**AUV6**	4000, 4000, 4000	2872,3498,3989	1053,1441,1160

### Simulation on the heterogeneous USV-AUV MASs obstacle avoidance

It is assumed that the heterogeneous USV-AUV formation has been generated before encountering obstacles. The initial state of the USV is (−4,11,7.7), the goal point of the USV is (23,−5.5,7.5). Dynamic obstacle speed is 0.2*m*/*s*. The parameters for IAPF are set in Table 3. The obstacles such as reefs and bridge pier are considered in simulation.

**Table 3:** The parameters for IAPF.

*d*_*o*_	*m*	*k*_*r*_	*k*_*a*1_	*k*_*a*2_	*ε*	*γ*	*δ*_*t*_
8	0.51	1.2	1	3	0.1	350	0.01

**Fig 12:**
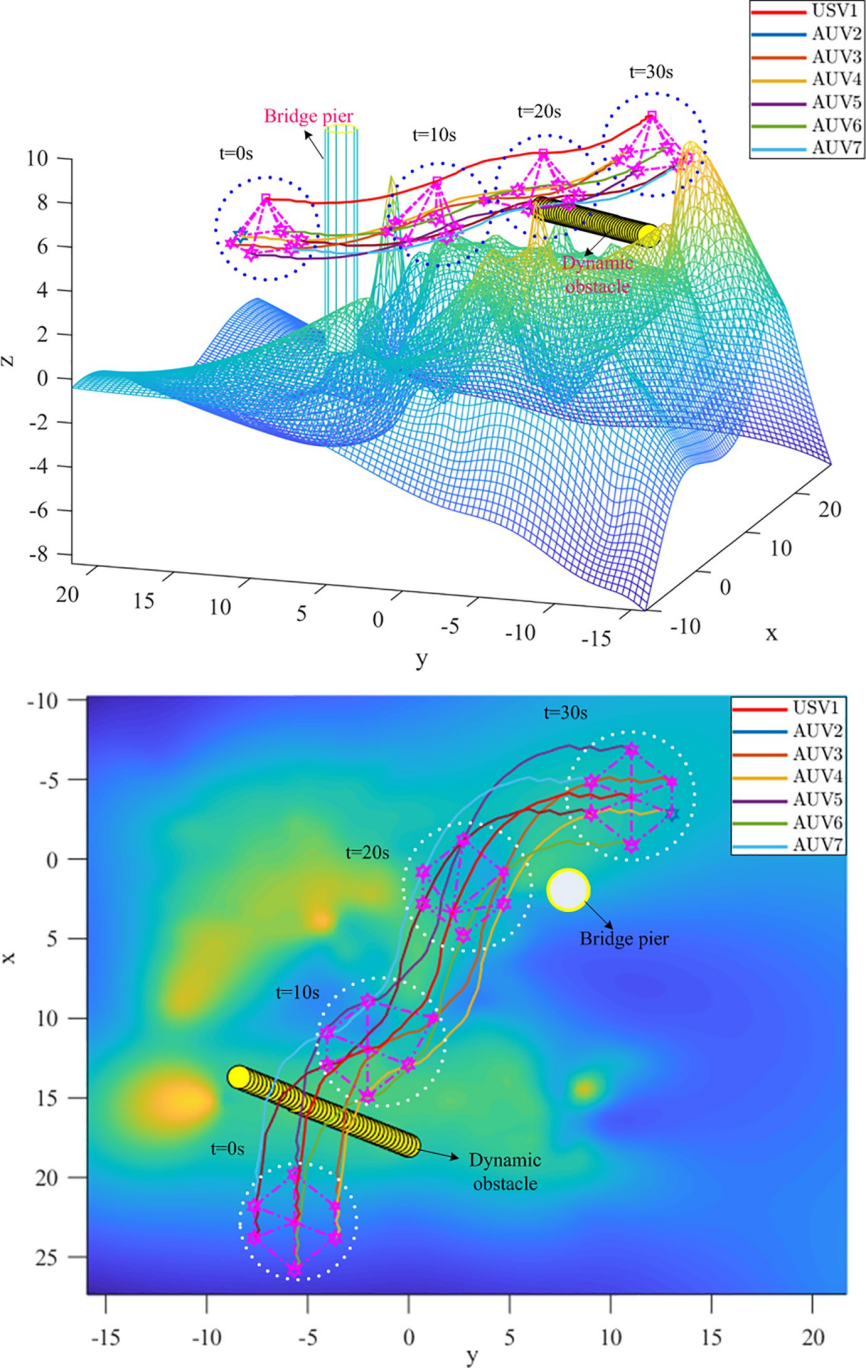
(a). The Path of the Heterogeneous USV-AUV Formation Using APF. (b). The Path of the Heterogeneous USV-AUV Formation Using APF in x-y direction.

**Fig 13:**
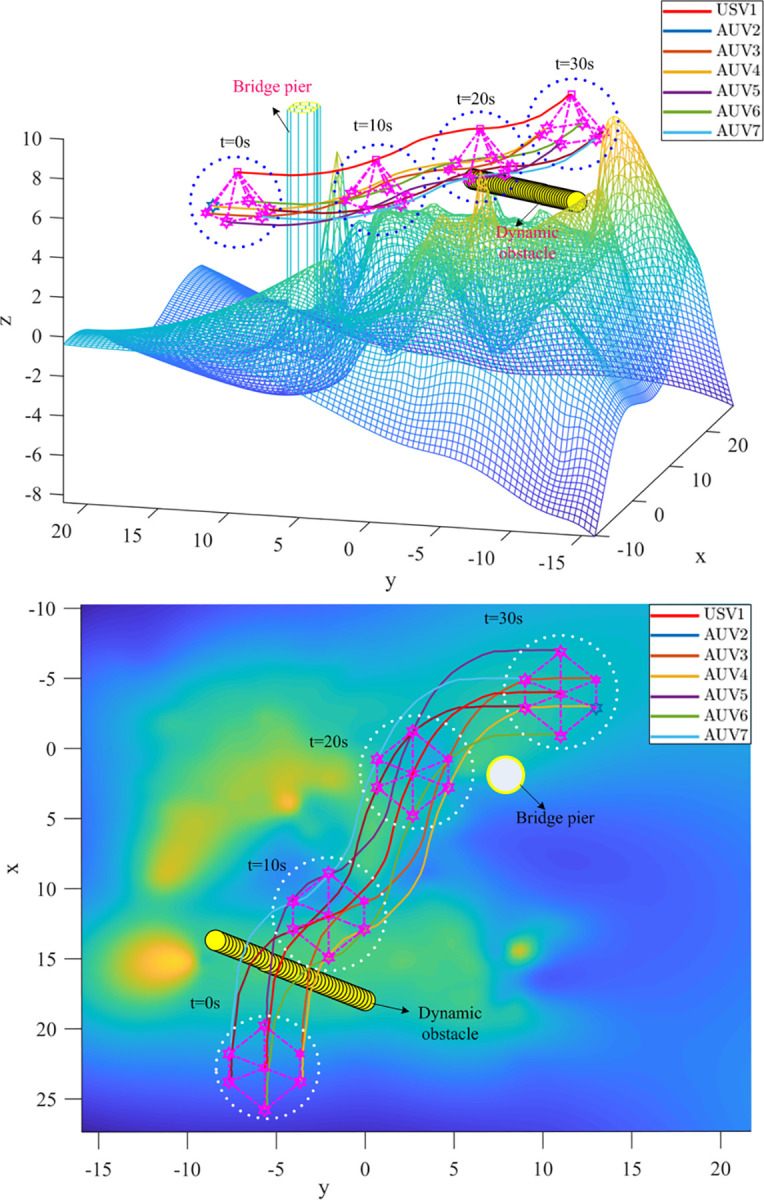
(a). The Path of the Heterogeneous USV-AUV Formation Using IAPF. (b). The Path of the Heterogeneous USV-AUV Formation Using IAPF in x-y direction.

Figs 12 and 13 show the path of the heterogeneous USV-AUV formation guided using APF and IAPF, respectively. Based on these images, the formation successfully avoids the obstacles and successfully reaches the target point. As can be seen in Fig 12, some agents dropped out or left the formation as they approached the target point. This indicates that there is a problem in the coordination among the formation members when using APF guidance. In contrast, we can see in Fig 13 that there are no dropouts or departures between members of the heterogeneous USV-AUV formation when the IAPF algorithm is used. This indicates that coordinated actions of the formation can be achieved more efficiently. In summary, based on the observations in Figs 12 and 13, it can be concluded that by introducing the IAPF, the heterogeneous USV-AUV formation shows better coordination and anti-disturbance performance in a multi-obstacle environment and can successfully reach the target point without agents dropping out or leaving the formation. This indicates that the proposed IAPF algorithm exhibits promising practical potential, and can provide an effective guidance method for performing heterogeneous formation tasks.

## Section 5: Conclusion

In this paper, an IAPF-based fixed-time ETC is proposed for handling heterogeneous USV-AUV multi-intelligent systems with input delays and uncertain interference. The algorithm operates under fixed-time ETC to reach consensus in a limited time and reduce communication energy consumption under any initial conditions. The IAPF algorithm is used to plan obstacle avoidance path for heterogeneous USV-AUV systems. The efficacy of the proposed method is validated through simulation results. However, this paper does not consider the communication problems between agents, such as noise and sampling rate, when considering heterogeneous USV-AUV formation control. These factors may have an impact on the performance of the algorithm. Therefore, future research can consider incorporating the modeling and treatment of the communication problem in the algorithm to improve the robustness and reliability of the algorithm. Furthermore, this paper also mentions conducting pool experiments to further validate the proposed algorithm. The actual physical experiments allow a more comprehensive evaluation of the performance and applicability of the algorithm and practical validation of the algorithm. In conclusion, the algorithm proposed in this paper with potential in handling heterogeneous USV-AUV multi-intelligent systems with input delays and uncertainty disturbances. Future research can further refine the algorithm by considering more communication factors and conducting practical experiments to validate the feasibility and effectiveness of the algorithm.

## Supporting information

S1 File (ZIP)
