# Supplementary figures and images for: Fixed-time event-triggered control for multi-agent systems with input delay
Source: PLoS One. 2023 Nov 13;18(11):e0293424. doi: 10.1371/journal.pone.0293424 (PMC10642835; doi:10.1371/journal.pone.0293424)

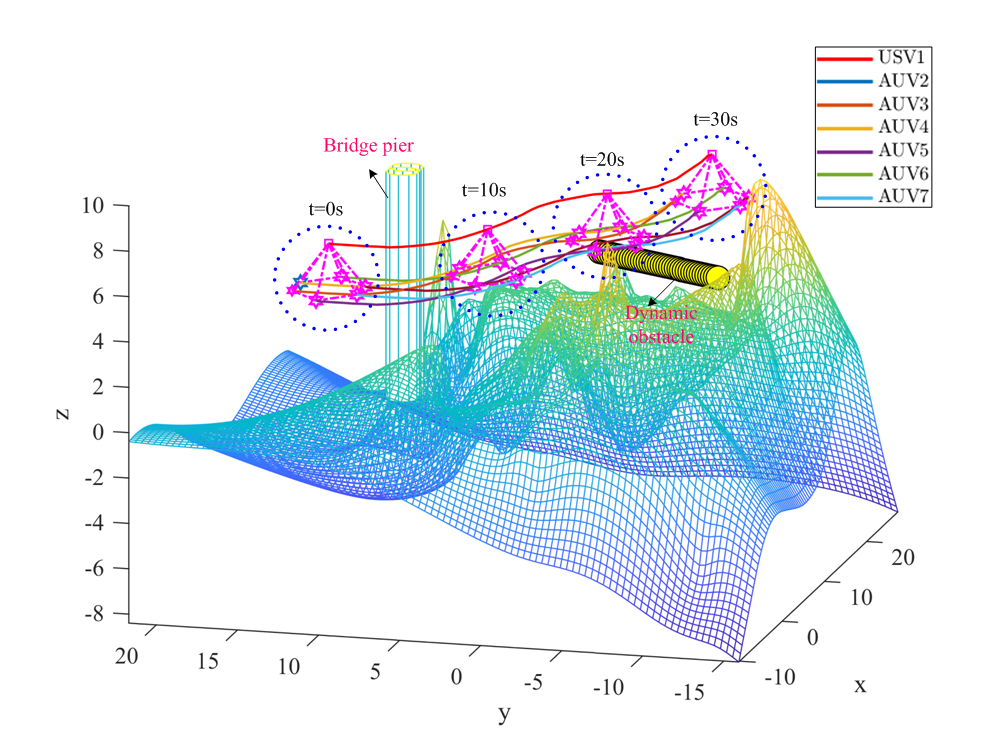

Supplement: S1 File — (ZIP) [file pone.0293424.s001.zip › Supporting Information/Fig. 13(a).tif]

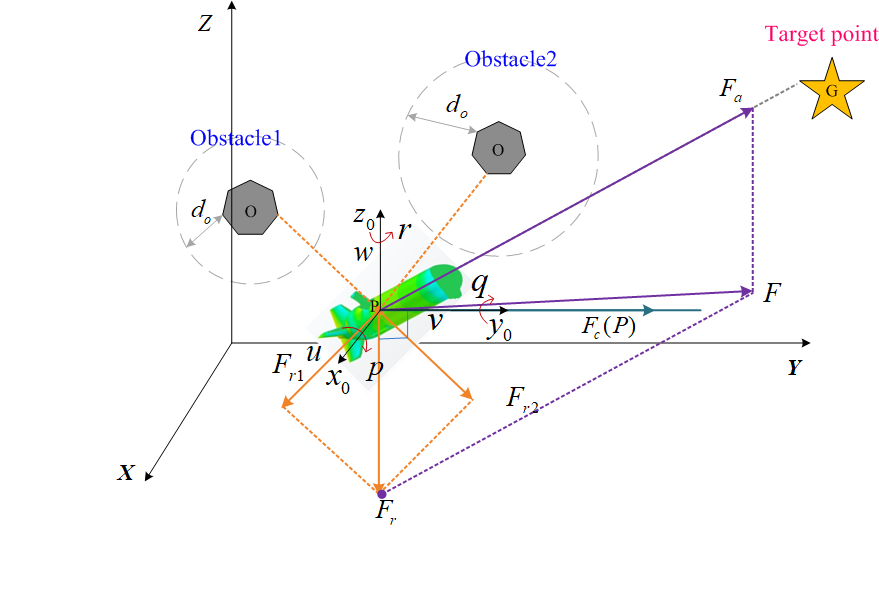

Supplement: S1 File — (ZIP) [file pone.0293424.s001.zip › Supporting Information/Fig.1.tif]

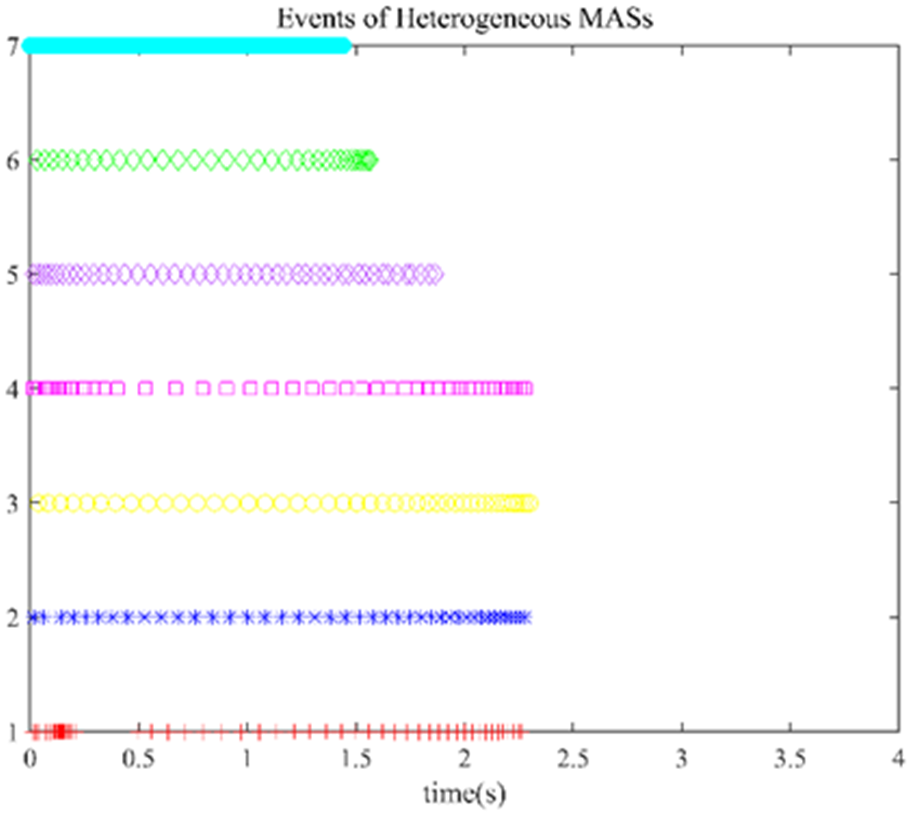

Supplement: S1 File — (ZIP) [file pone.0293424.s001.zip › Supporting Information/Fig.10.tif]

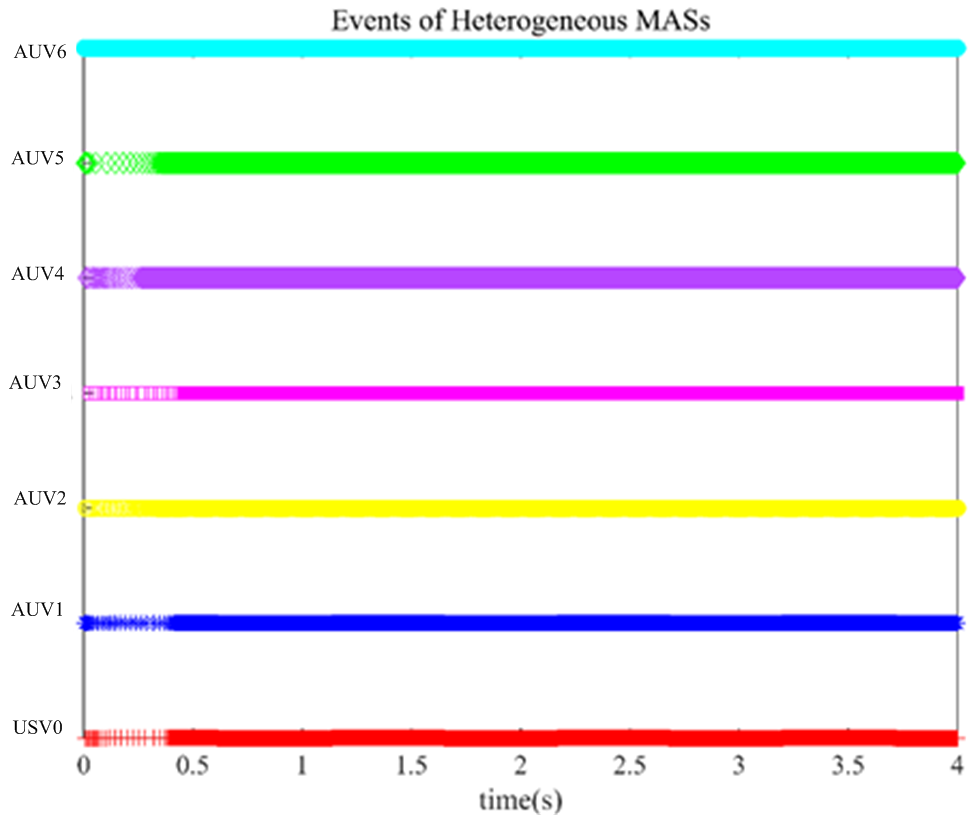

Supplement: S1 File — (ZIP) [file pone.0293424.s001.zip › Supporting Information/Fig.11.tif]

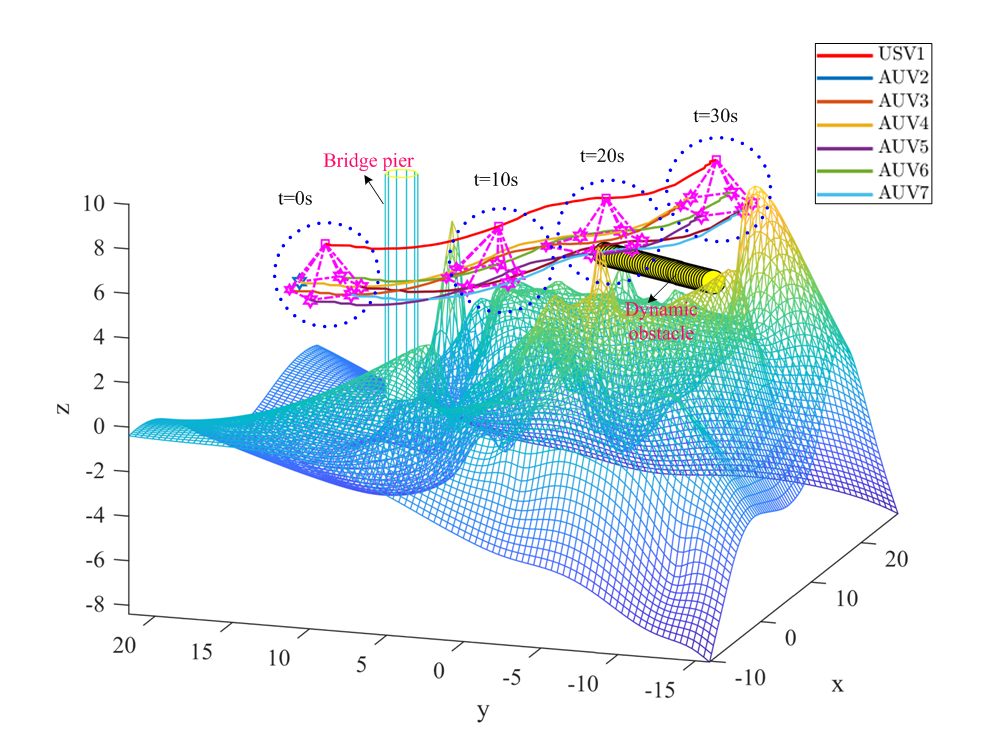

Supplement: S1 File — (ZIP) [file pone.0293424.s001.zip › Supporting Information/Fig.12(a).tif]

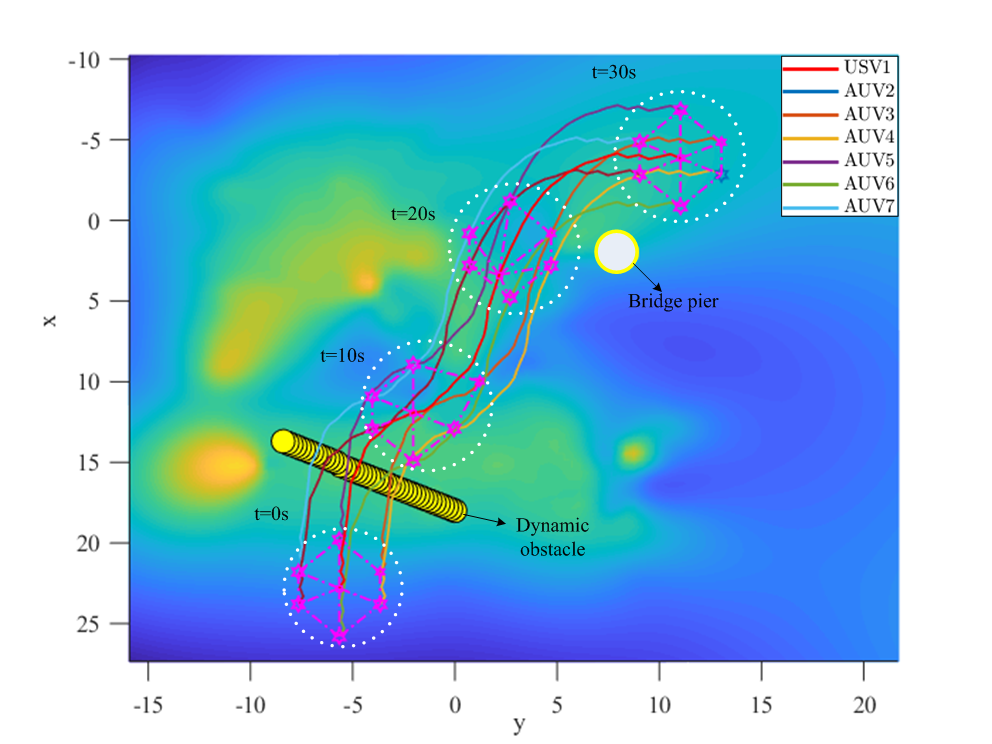

Supplement: S1 File — (ZIP) [file pone.0293424.s001.zip › Supporting Information/Fig.12(b).tif]

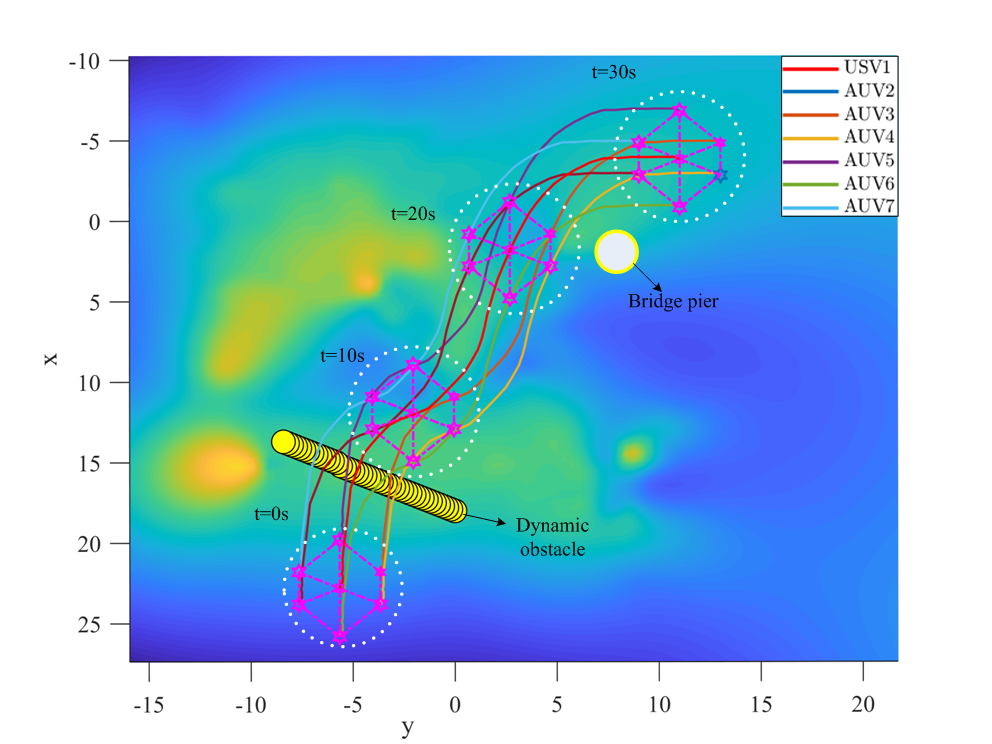

Supplement: S1 File — (ZIP) [file pone.0293424.s001.zip › Supporting Information/Fig.13(b).tif]

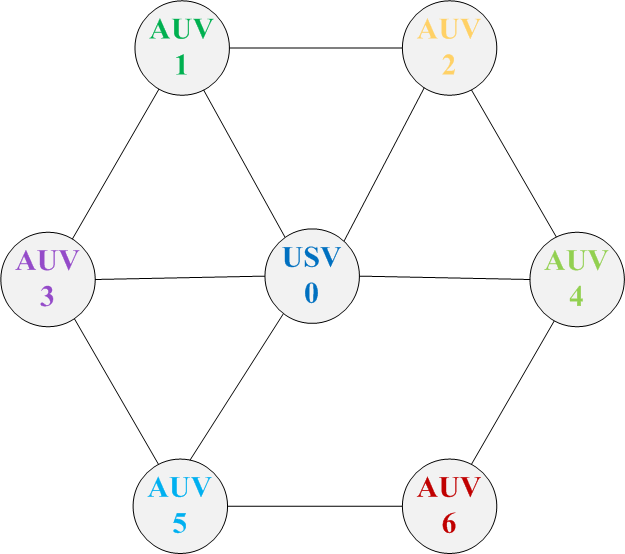

Supplement: S1 File — (ZIP) [file pone.0293424.s001.zip › Supporting Information/Fig.2.tif]

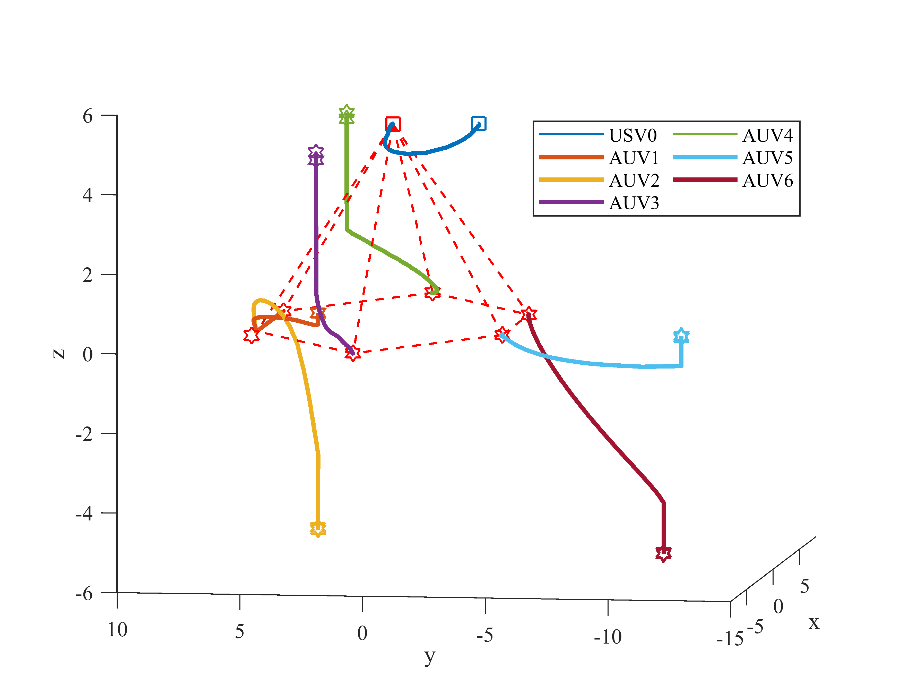

Supplement: S1 File — (ZIP) [file pone.0293424.s001.zip › Supporting Information/Fig.3.tif]

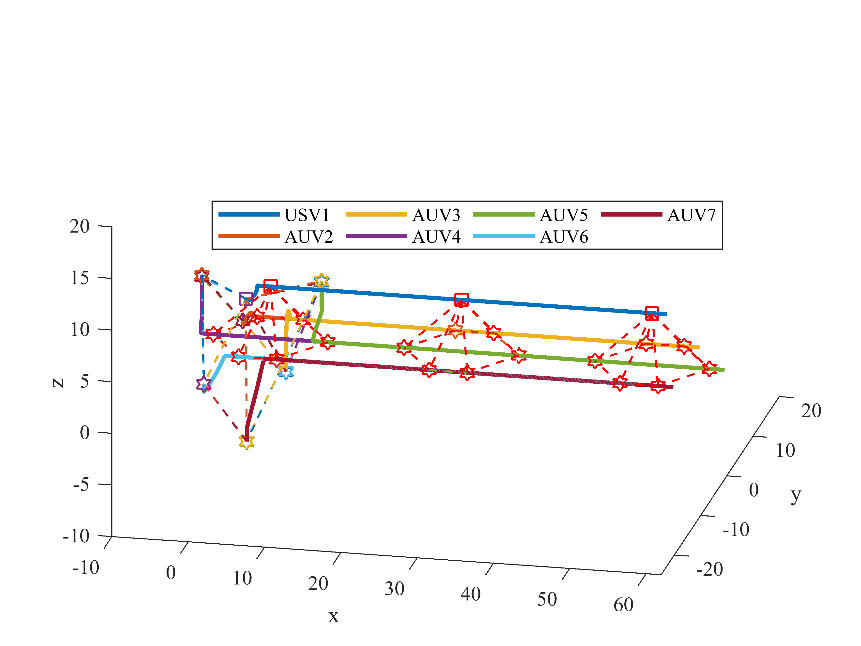

Supplement: S1 File — (ZIP) [file pone.0293424.s001.zip › Supporting Information/Fig.4.tif]

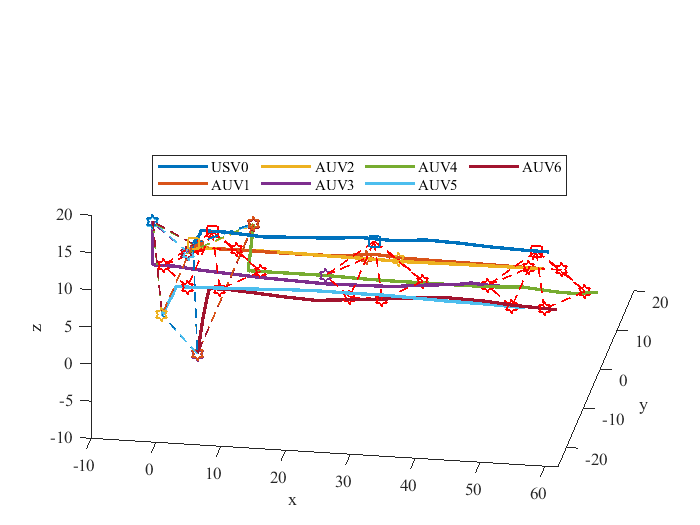

Supplement: S1 File — (ZIP) [file pone.0293424.s001.zip › Supporting Information/Fig.5.tif]

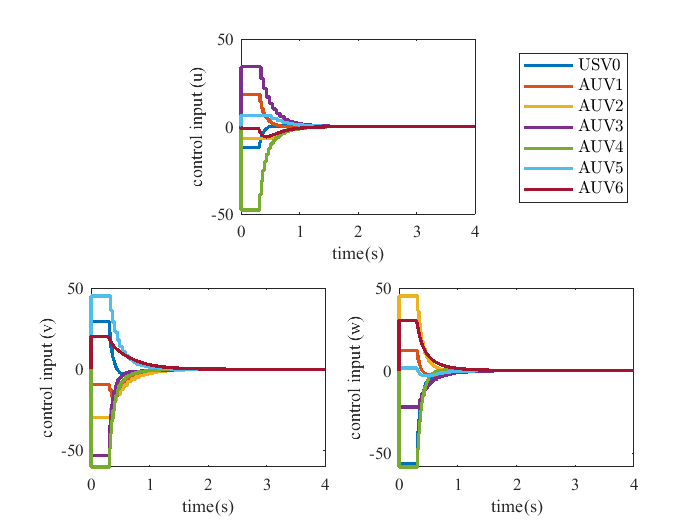

Supplement: S1 File — (ZIP) [file pone.0293424.s001.zip › Supporting Information/Fig.6.tif]

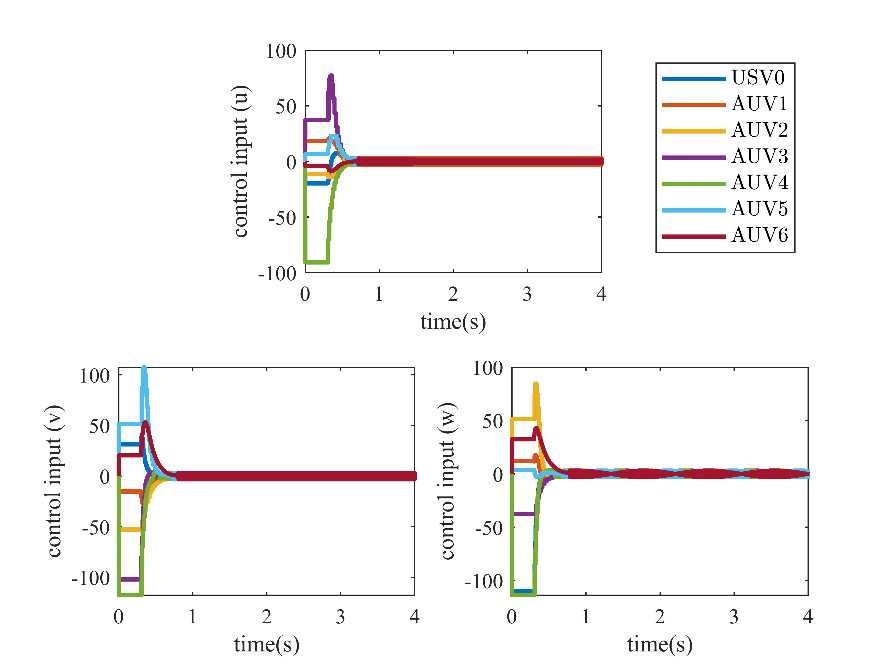

Supplement: S1 File — (ZIP) [file pone.0293424.s001.zip › Supporting Information/Fig.7.tif]

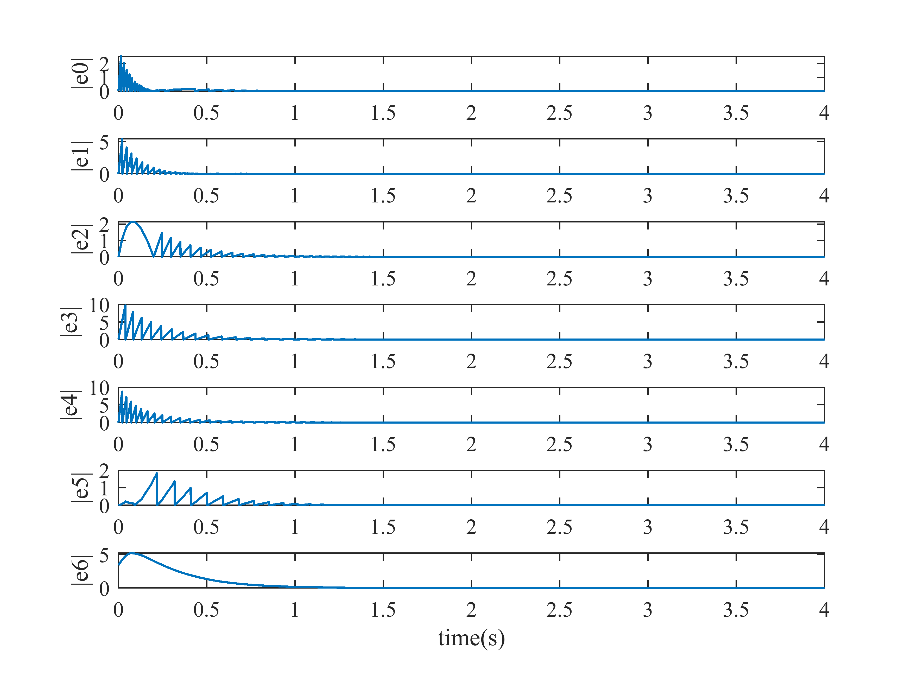

Supplement: S1 File — (ZIP) [file pone.0293424.s001.zip › Supporting Information/Fig.8(a).tif]

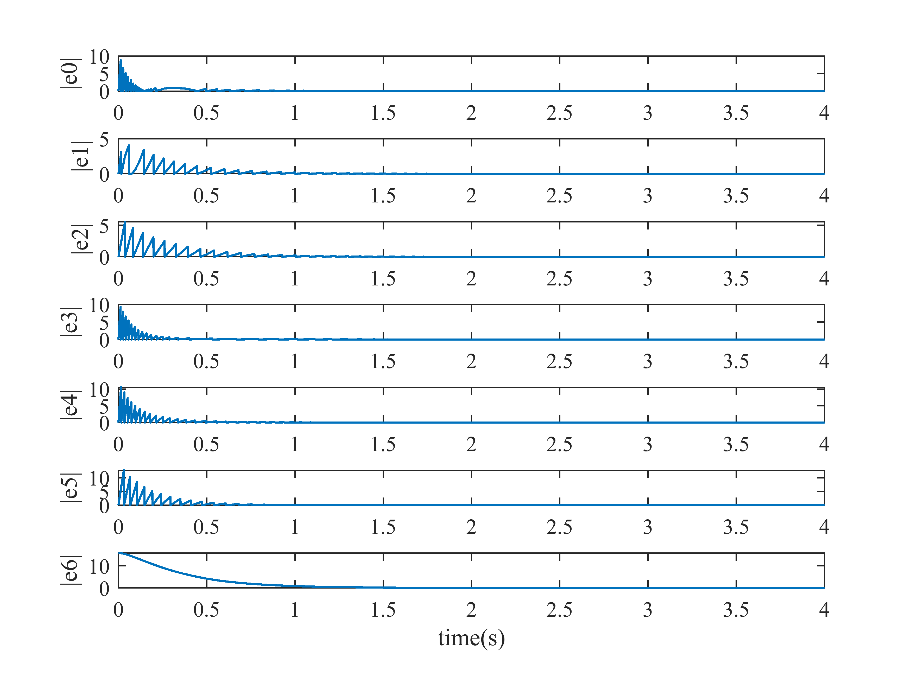

Supplement: S1 File — (ZIP) [file pone.0293424.s001.zip › Supporting Information/Fig.8(b).tif]

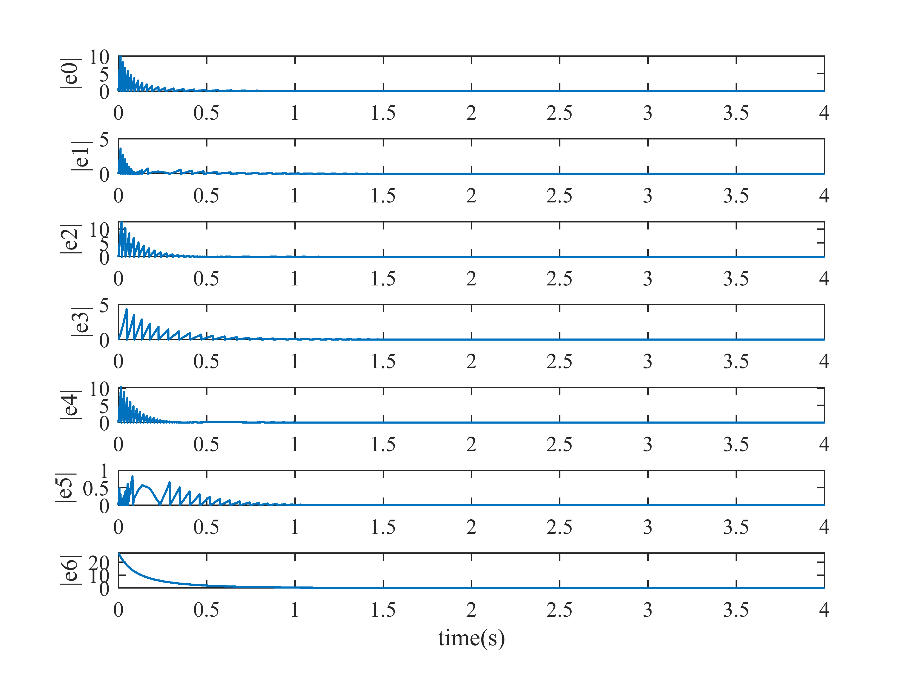

Supplement: S1 File — (ZIP) [file pone.0293424.s001.zip › Supporting Information/Fig.8(c).tif]

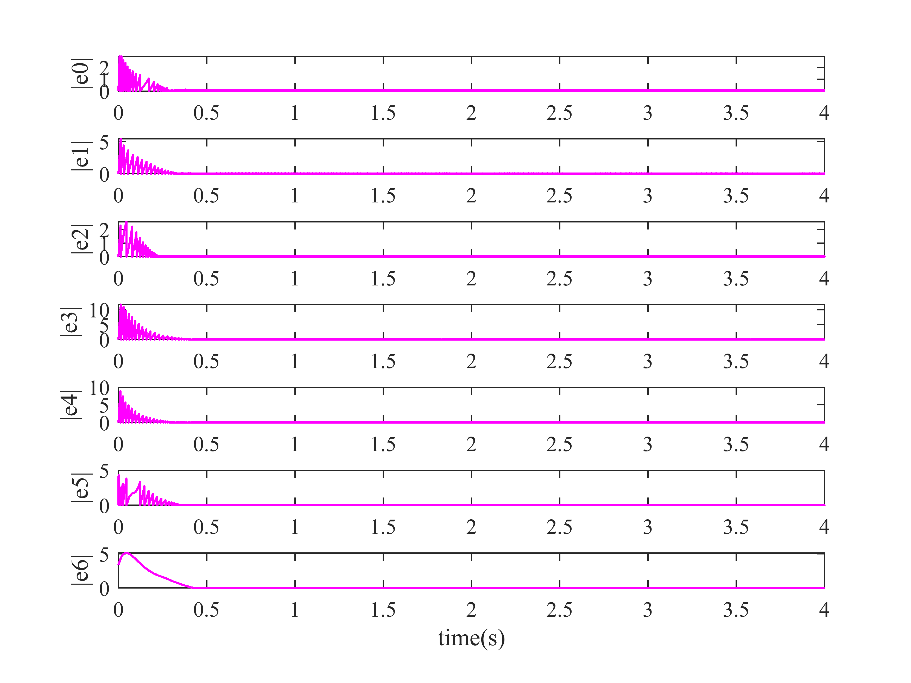

Supplement: S1 File — (ZIP) [file pone.0293424.s001.zip › Supporting Information/Fig.9(a).tif]

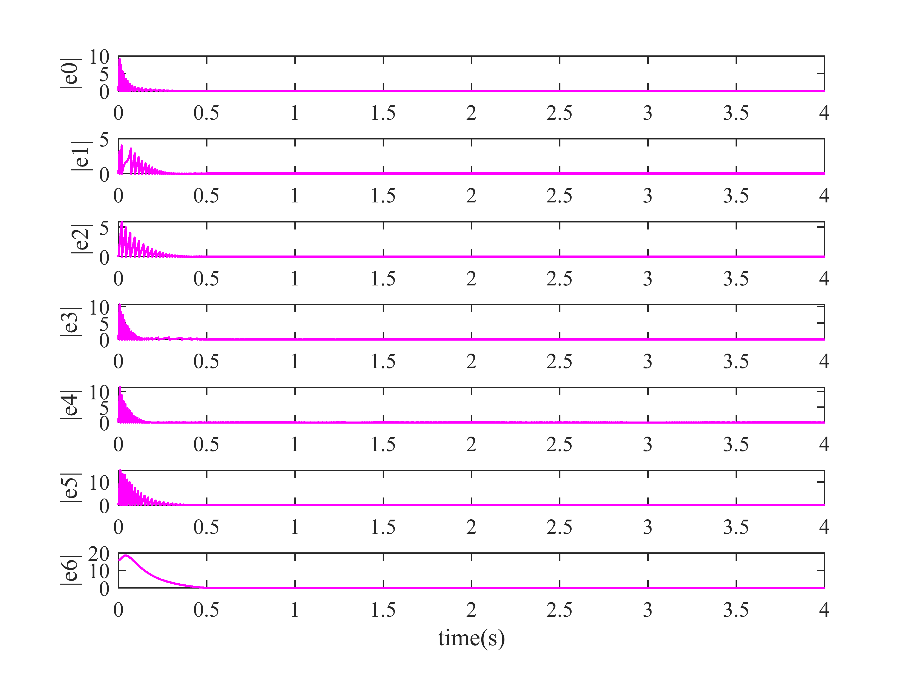

Supplement: S1 File — (ZIP) [file pone.0293424.s001.zip › Supporting Information/Fig.9(b).tif]

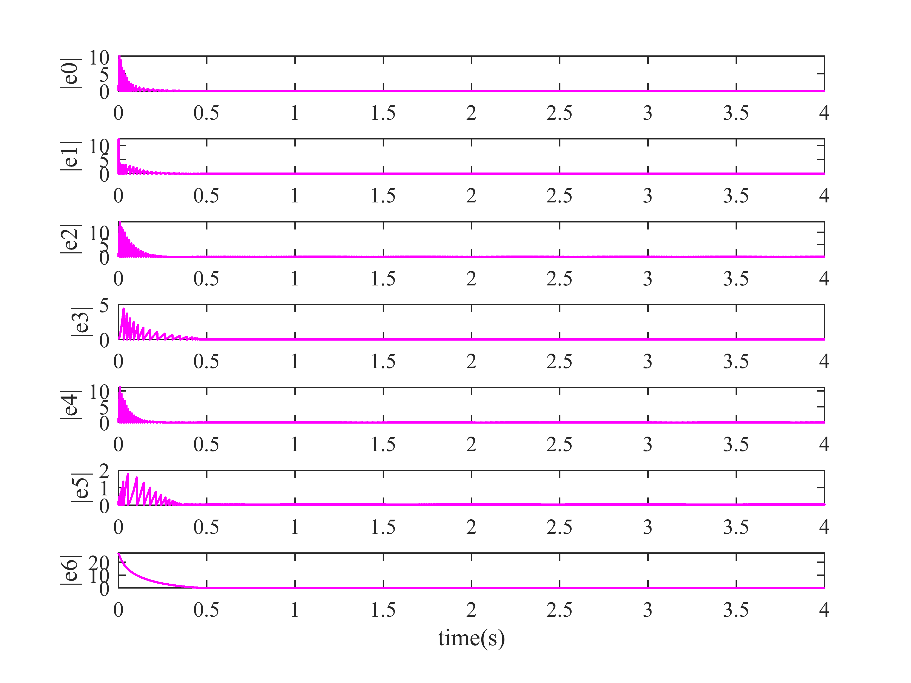

Supplement: S1 File — (ZIP) [file pone.0293424.s001.zip › Supporting Information/Fig.9(c).tif]
