# Supplementary material for: Fixed-time event-triggered control for multi-agent systems with input delay
Source: PLoS One. 2023 Nov 13;18(11):e0293424. doi: 10.1371/journal.pone.0293424 (PMC10642835; doi:10.1371/journal.pone.0293424)
Supplement: S1 File — (ZIP) [file pone.0293424.s001.zip › Supporting Information/Table.doc]

**Table 1. T****he State of the Agents.**

|  | **USV0** | **AUV1** | **AUV2** | **AUV3** | **AUV4** | **AUV5** | **AUV6** |
| --- | --- | --- | --- | --- | --- | --- | --- |
| **x** | 0.5 | -2 | 3 | -8 | 7.5 | -3 | 2.5 |
| **y** | -3 | 3 | 5 | 2 | 4 | -12 | -10 |
| **z** | 5 | 0.5 | -5 | 5 | 4.5 | 1 | -6 |

**Table 2. The Times of Triggered for Each Agent.**

| **Agent** | **Time-triggered** | **Event-triggered**  **with sign controller (17)** | **Event-triggered**  **with sat controller (29)** |
| --- | --- | --- | --- |
| **USV0** | 4000, 4000, 4000 | 1875,1812,1824 | 47,56,46 |
| **AUV1** | 4000, 4000, 4000 | 1849,1841,1815 | 57,42,52 |
| **AUV2** | 4000, 4000, 4000 | 1903,1815,1876 | 37,44,61 |
| **AUV3** | 4000, 4000, 4000 | 1817,1782,1781 | 33,45,42 |
| **AUV4** | 4000, 4000, 4000 | 1861,1901,1885 | 45,46,52 |
| **AUV5** | 4000, 4000, 4000 | 1842,1810,1839 | 26,34,45 |
| **AUV6** | 4000, 4000, 4000 | 2872,3498,3989 | 1053,1441,1160 |

**Table 3. The Parameters for IAPF.**

| 8 | 0.51 | 1.2 | 1 | 3 | 0.1 | 350 | 0.01 |
| --- | --- | --- | --- | --- | --- | --- | --- |
